# Precision Temperature Control of Volume Phase Transition in Biocompatible PEG-Based Nanogels for Triggered Drug Release

**DOI:** 10.1021/acsnanomed.6c00111

**Published:** 2026-06-10

**Authors:** Sofia Patri, Saanchi Agrawal, Paul Joseph Kempen, Nguyen Thi Kim Thanh, Nazila Kamaly

**Affiliations:** † Department of Materials, Imperial College London, London SW7 2AZ, United Kingdom; ‡ Department of Chemistry, Imperial College London, London SW7 2AZ, United Kingdom; § National Center for Nano Fabrication and Characterization, Technical University of Denmark, Kgs Lyngby 2800, Denmark; ∥ Healthcare Biomagnetics and Nanomaterials Laboratories, University College London, London W1S 4BS, United Kingdom; ⊥ Biophysics Group, Department of Physics and Astronomy, 4919 University College London, London WC1E 6BT, United Kingdom

**Keywords:** Nanogels, thermoresponsive polymers, drug release, cancer therapy, stimuli responsive

## Abstract

Thermoresponsive nanogels (NGs) can reversibly alter their structure in response to temperature changes. This enables controlled drug release, targeted therapy, and improved treatment precision under physiological or externally applied thermal conditions. Despite these advantages, few studies have systematically tuned the volume phase transition temperature (VPTT) of oligo­(ethylene glycol) methacrylates (OEGMA)-based NGs across physiologically relevant temperatures while maintaining stability and biodegradability. In this work, NGs were synthesized using di­(ethylene glycol) methyl ether methacrylate (DEGMA), tri­(ethylene glycol) methyl ether methacrylate (TEGMA), and 2-methoxyethyl methacrylate (MOEMA) monomers, achieving finely tuned VPTT with a precision within 1 °C. The NGs exhibited diameters ranging from 200 to 350 nm and a negative ζ-potential. Upon reaching the VPTT, the NGs shrank to sizes below 100 nm while becoming monodisperse. Specifically, NG@TEGMA exhibited VPTT between 50–60 °C, NG@DEGMA between 30–45 °C, and NG@TEGMA_DEGMA between 64–66 °C, and NG@TEGMA_MOEMA with molar ratios of 40%, 30%, and 20% displayed VPTT of 37, 47, and 52 °C, respectively. NG@TEGMA NGs were further polymerized with a methacrylated docetaxel derivative, achieving an encapsulation efficiency of 93 ± 4%. These NGs released approximately 90% of the drug upon reaching the VPTT, while nonthermoresponsive controls only released about 20% of the drug. Furthermore, NG@TEGMA demonstrated negligible cytotoxicity and efficient cellular uptake. This study demonstrates a biocompatible and degradable platform of OEGMA-based thermoresponsive NGs for biomedical applications such as hyperthermia-driven drug delivery for cancer therapy with superior thermoresponsive behavior to previously reported OEGMAs-, NIPAM-, or VCL-based NGs.

## Graphical abstract

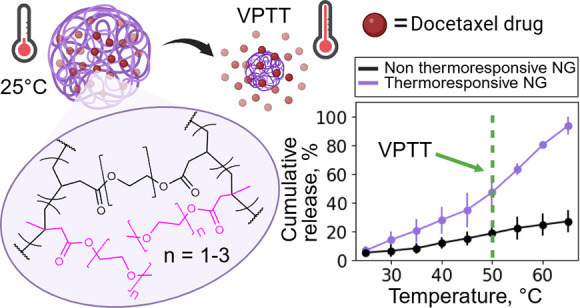

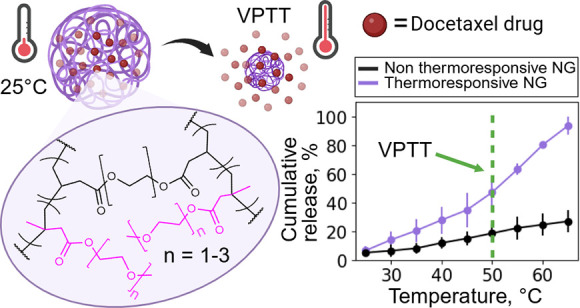

## Introduction

Stimuli-responsive nanogels (NGs) are a promising class of soft nanoparticles for biomedical applications. Composed of covalently cross-linked polymer chains swollen with water, NGs can undergo controlled conformational changes in response to specific stimuli, enabling precise release of encapsulated therapeutic payloads such as small-molecule drugs or proteins.^1,2^ These properties make NGs attractive candidates for targeted drug delivery and vaccine development.^1^ Common stimuli, for which the corresponding cleavable or reactive bonds reside within the nanogels, include pH shifts,^3,4^ redox processes^5,6^ and enzymatic activity.^7,8^ Among stimuli-responsive NGs, thermoresponsive systems are particularly appealing due to the widespread use of heat in biomedical treatments such as hyperthermia-assisted chemotherapy (therapeutic range 39–45 °C) and ultrasound focal therapy (therapeutic range 55–70 °C).^9–11^ Thermoresponsive behavior arises from the balance between hydrophilic and hydrophobic segments in the polymer network. Below a characteristic temperature, the network is hydrated and swollen; above the volume phase transition temperature (VPTT), it collapses, expelling water and any encapsulated cargo.^2,9^ While the VPTT is related to the lower critical solution temperature (LCST) of bulk polymers, it can differ by several degrees, as it is also influenced by factors such as cross-linking density, NG size, and surface properties.^12^ Several monomers have been used to design thermoresponsive NGs. *N*-Isopropylacrylamide (NIPAM) is among the most widely used monomers, as poly­(*N*-isopropylacrylamide) (*p*NIPAM) exhibits well-characterized thermoresponsive behavior, with a bulk LCST of approximately 32 °C.^12^ However, its clinical use is limited by its cytotoxicity, poor biodegradability, and a VPTT below the physiological range.^11,13,14^ Vinyl caprolactam (VCL) offers improved biocompatibility and a polymer with LCST above 38 °C,^15^ yet it remains nonbiodegradable.^1,12,16^ Poly­(ethylene glycol) (PEG) derivatives, particularly oligo­(ethylene glycol) methacrylates (OEGMA), combine excellent biocompatibility^17,18^ with tunable and reversible thermoresponsive behavior,^19^ making them superior alternatives to NIPAM- or VCL-based systems.^17,18,20^ The LCST of OEGMA polymers can be precisely adjusted by varying side-chain length or copolymer composition, providing unique control over the temperature responsivity.^17,20–23^ Despite these advantages, few studies have systematically tuned the VPTT of OEGMA-based NGs across physiologically relevant temperatures while maintaining stability, biodegradability, and drug delivery functionality. For instance, Kong and colleagues reported OEGMA NGs with a VPTT of 37 °C at pH 10, but external factors such as pH and solvent affected the transition.^24^ In 2021, Zhang et al. achieved NGs with a VPTT of 36 °C using di­(ethylene glycol) methyl ether methacrylate (DEGMA) and poly­(ethylene glycol)­methyl ether methacrylate (PEGMA), but the conformational change occurs gradually over several °C, limiting the control over the shrinking-swelling process.^25^ Biglione et al. incorporated multiple thermoresponsive OEGMAs monomers into NGs, but swelling above the cloud point and aggregation above 60 °C compromised drug delivery performance.^26^ More recently, Macchione et al. reported NGs with VPTT of 39–50 °C, still within a limited temperature range.^27^

Herein, we present a strategy to fine-tune the VPTT of OEGMA-based thermoresponsive NGs. Tri­(ethylene glycol) methyl ether methacrylate (TEGMA) and DEGMA were selected as thermoresponsive monomers, with acrylamide as a comonomer and PEG diacrylate (PEGDA, MW 250) as the cross-linker. By varying monomer combinations and incorporating two thermoresponsive units simultaneously, NGs with VPTT ranging from 30–45 °C (NG@DEGMA) and 50–60 °C (NG@TEGMA) were obtained. The introduction of the hydrophobic comonomer 2-methoxyethyl methacrylate (MOEMA) further modulated the NG@TEGMA’s transition temperature. The physicochemical properties of the NGs, including particle size, VPTT, stability under long-term storage at 4 °C and degradability at 37 °C, were comprehensively characterized. Additionally, a methacrylated docetaxel derivative (**1**) was polymerized into NG@TEGMA to evaluate drug release, demonstrating proof-of-concept for drug delivery applications. Finally, cytotoxicity and cellular uptake studies confirmed the biocompatibility of the prepared NGs.

In this work, we establish a versatile platform for precisely tuning the VPTT of biocompatible, degradable OEGMA-based NGs across physiologically relevant temperature ranges, while preserving structural stability and demonstrating their potential for targeted drug delivery.

## Results and Discussion

### Nanogel Synthesis

Polymers based on DEGMA and TEGMA, shown in Figure S1, are known to be thermoresponsive, with reported LCSTs of 26 and 52 °C, respectively.^28,29^ DEGMA was selected to provide a lower transition point, while TEGMA extended the range to higher temperatures. It is well established that the molar mass, the number of ethylene glycol (EG) units and the distal end functional group of the monomer can affect the cloud point and LCST of bulk polymers.^21^ In particular, an increased number of EG units enhances the hydrophilicity of the polymer, which results in a higher cloud point.^21^ To further modulate the VPTT, MOEMA, a hydrophobic monomer lacking a LCST,^29^ was incorporated as a comonomer. This approach allowed the preparation of NGs with customized thermoresponsive properties spanning a wide range of temperatures.

The thermoresponsive NGs were synthesized using a modified method inspired by Dabas et al.^30^ All the required monomers (acrylamide, PEGDA cross-linker and OEGMA monomers) were dissolved in distilled (DI) water at RT under inert conditions, followed by the addition of the surfactant sodium dodecyl sulfate (SDS) and cooling to 0 °C. Polymerization was initiated using *N,N,N′,N′*-tetramethylethylenediamine (TEMED) base and ammonium persulfate (APS) as the initiator and the NGs were allowed to form over 1 h under fully aqueous conditions. The reaction scheme is shown in Figure 1 and experimental details are in the methods section. The resulting NGs were purified using centrifugation. Details of the different formulations for the samples are given in Table S1.

**Figure 1:**
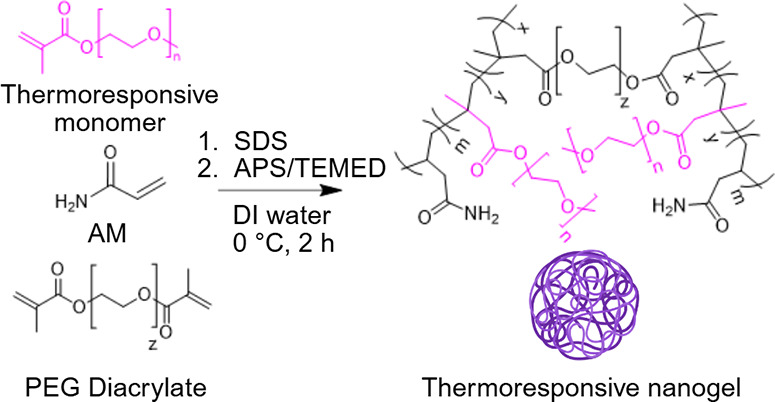
General NG synthesis scheme in distilled (DI) water incorporating the thermoresponsive monomers (MOEMA *n* = 1; DEGMA *n* = 2; TEGMA *n* = 3). Abbreviations: AM = acrylamide, PEG diacrylate = polyethylene glycol diacrylate 250 MW, SDS = sodium dodecyl sulfate, APS = ammonium persulfate, TEMED = *N,N,N′,N′*-tetramethylethylenediamine.

This synthesis method was rapid and straightforward, with the reaction being completed in under 2 h and purification via centrifugation requiring only 45 min. Compared with approaches that rely on organic solvents, prolonged reaction or dialysis times,^31^ or ultrasonication,^26^ this method offered a more practical and scalable route suitable for biomedical applications.

Cross-linking of the NGs was confirmed by infrared spectroscopy. The spectra of the starting monomers, Figure S2, display a characteristic absorption band at 1636 cm^–1^, corresponding to the C=C stretching vibration of the methacrylate/acrylate groups. This band was absent in the spectra of the NGs, Figures S3–S7, which indicated successful consumption of the vinyl groups during polymerization. In contrast, absorption bands at 2916 and 2851 cm^–1^ were attributed to aliphatic C–H stretching vibrations and a strong band at 1725 cm^–1^ was assigned to ester carbonyl stretching. Both absorption bands confirmed the formation of the cross-linked polymer network.^26^

Figure 2 and Tables S1 and S2 summarize the size, PDI and ζ-potential data for all the formulations of thermoresponsive NGs investigated. The diameters after synthesis were consistent with values previously reported for NGs prepared using comparable formulations or alternative monomers.^15,25,27^ Most NGs showed a large PDI, indicating a low degree of monodispersity in the samples.^32^ However, elevated PDIs are commonly observed in methacrylate polymerization^1,33^ and are typical for OEGMA- or dextran-methacrylate–based NGs.^15,25,31^ From a pharmaceutical perspective, NGs should ideally be as monodisperse as possible,^34^ although previous studies have demonstrated that higher PDIs do not compromise their applicability.^35^ The negative ζ-potentials, ranging from −17 ± 7 (NG@ TEGMA_MOEMA_2) to −36 ± 2 (NG@DEGMA_1) were in accordance with those reported previously in the literature.^8^ They were sufficiently negative to ensure colloidal stability, as charged NGs are less likely to aggregate even in the presence of serum proteins.^5^

**Figure 2:**
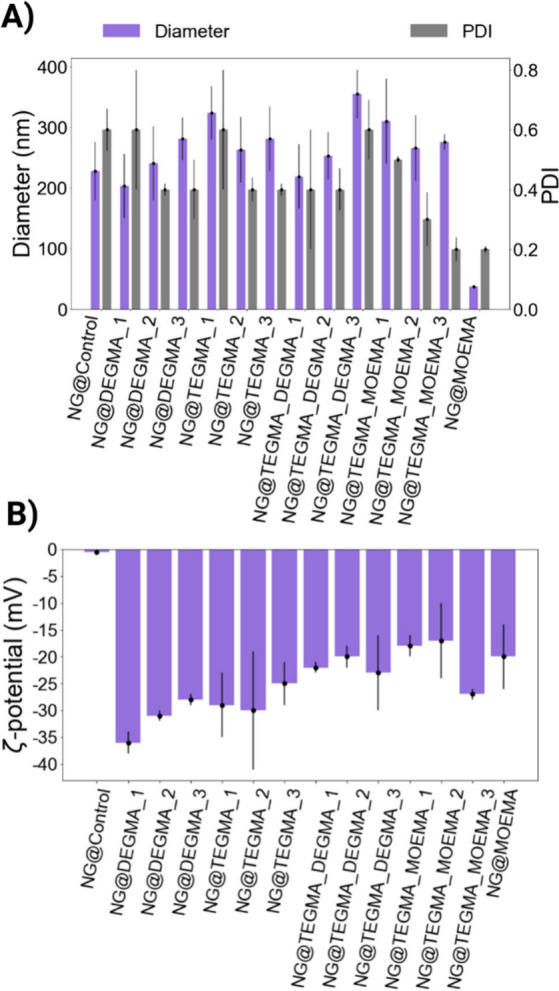
Characterization of NGs after synthesis in water at 25 °C: (A) Hydrodynamic diameter and polydispersity (PDI) as obtained from dynamic light scattering (DLS) measurements. (B) ζ-potential (mV) as obtained from electrophoretic scattering measurements (measured in water, pH 7.2). The samples are named after the thermoresponsive monomers used. The subscripts in the sample’s names are used to distinguish formulations with the same monomers but different molar quantities, as detailed in Table S1. In all cases, data are presented as mean ± standard deviation for *n* = 3.

### Characterization of the VPTT

The VPTT was determined from size vs temperature measurements with dynamic light scattering (DLS). Briefly, the NGs’ size was monitored over a temperature range of 25–80 °C, with 5 °C increments and 120 s of equilibration. The measurements revealed a reduction in NG size, as summarized in Figure 3A-D. An example of the DLS data is shown in Figure 3E. Once the transition region was identified, measurements were repeated at 1 °C intervals within a 10 °C range to determine the precise VPTT.

**Figure 3:**
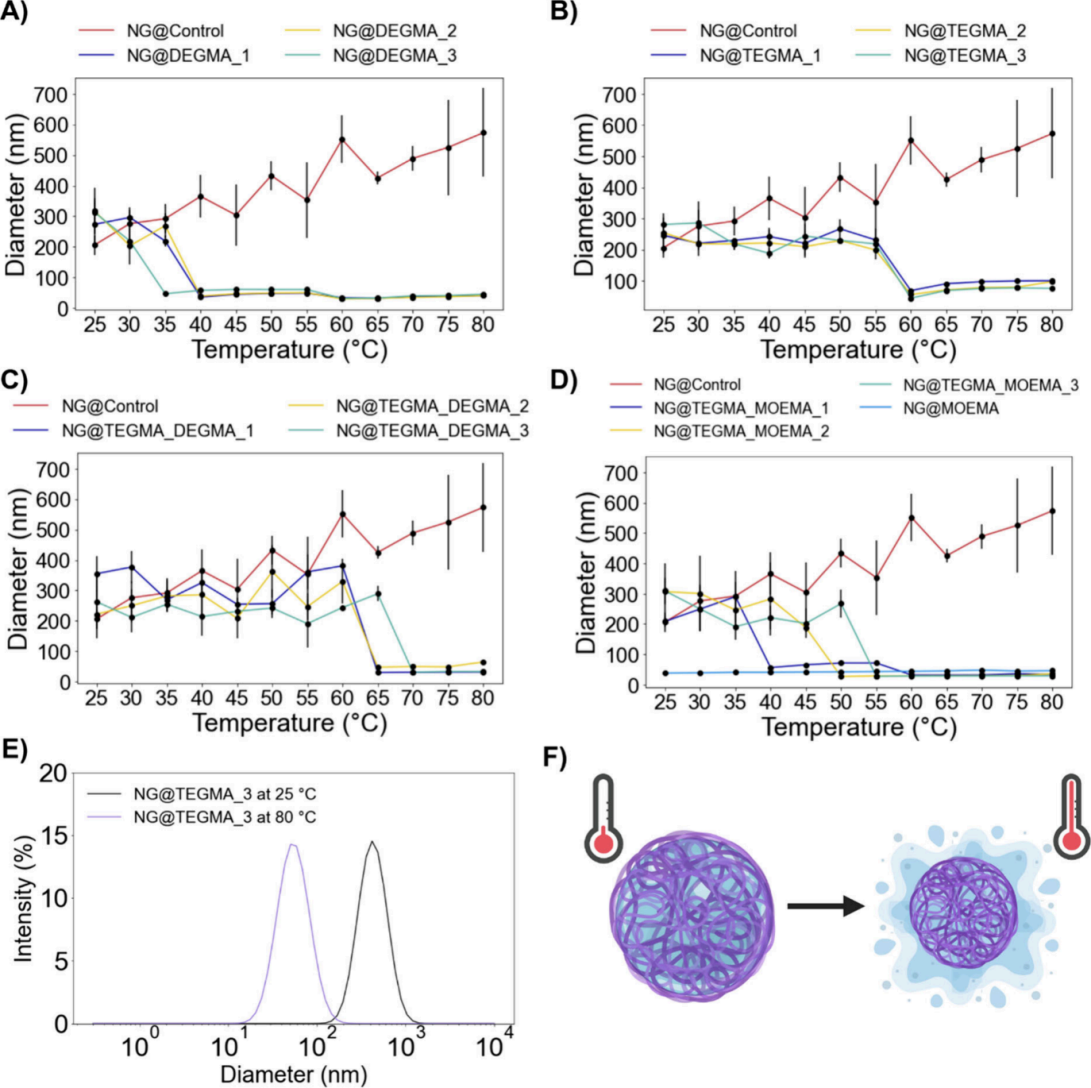
Characterization of volume phase transition temperature (VPTTs) (hydrodynamic diameter vs temperature measurements) in water for (A) NG@Control and NG@DEGMA series, (B) NG@Control and NG@TEGMA series, (C) NG@Control and NG@TEGMA_DEGMA series, and (D) NG@Control, NG@TEGMA_MOEMA series, and NG@MOEMA. (E) Representative intensity weighted size distribution before and after reaching the VPTT for NG@TEGMA_3. (F) Schematic illustration of the conformational change of the NGs due to the VPTT. In all cases, data are presented as mean ± standard deviation for *n* = 3.

Pure NG@DEGMA NGs exhibited VPTT between 30 and 45 °C, whereas pure NG@TEGMA NGs showed VPTT between 50 and 60 °C. Both NG@DEGMA and NG@TEGMA NGs shrank at the VPTT with a precision of 1 °C, Figure 4A–F, decreasing from 250/300 nm to below 80 nm. The conformational change at the VPTT is an entropy-driven process: hydrogen bonding between the polymer matrix and the solvent is weakened due to the unfavorable entropy of mixing, which causes the polymer to dissociate from water.^36^ This results in the transition from a swollen state below the VPTT to a collapsed state above it. The cryo-TEM images, Figure 4Gand H, confirmed the shrinking-swelling behavior of the thermoresponsive NGs formulations. The thermoresponsive NGs NG@TEGMA exhibited a pronounced reduction in diameter at their VPTT. The same experiment was performed with negative stained samples shown in Figure S12, which also demonstrates that the diameter is fully recovered after 24 h. For the control NGs, the particle diameter remained unchanged across all temperatures, indicating no thermal response.

**Figure 4:**
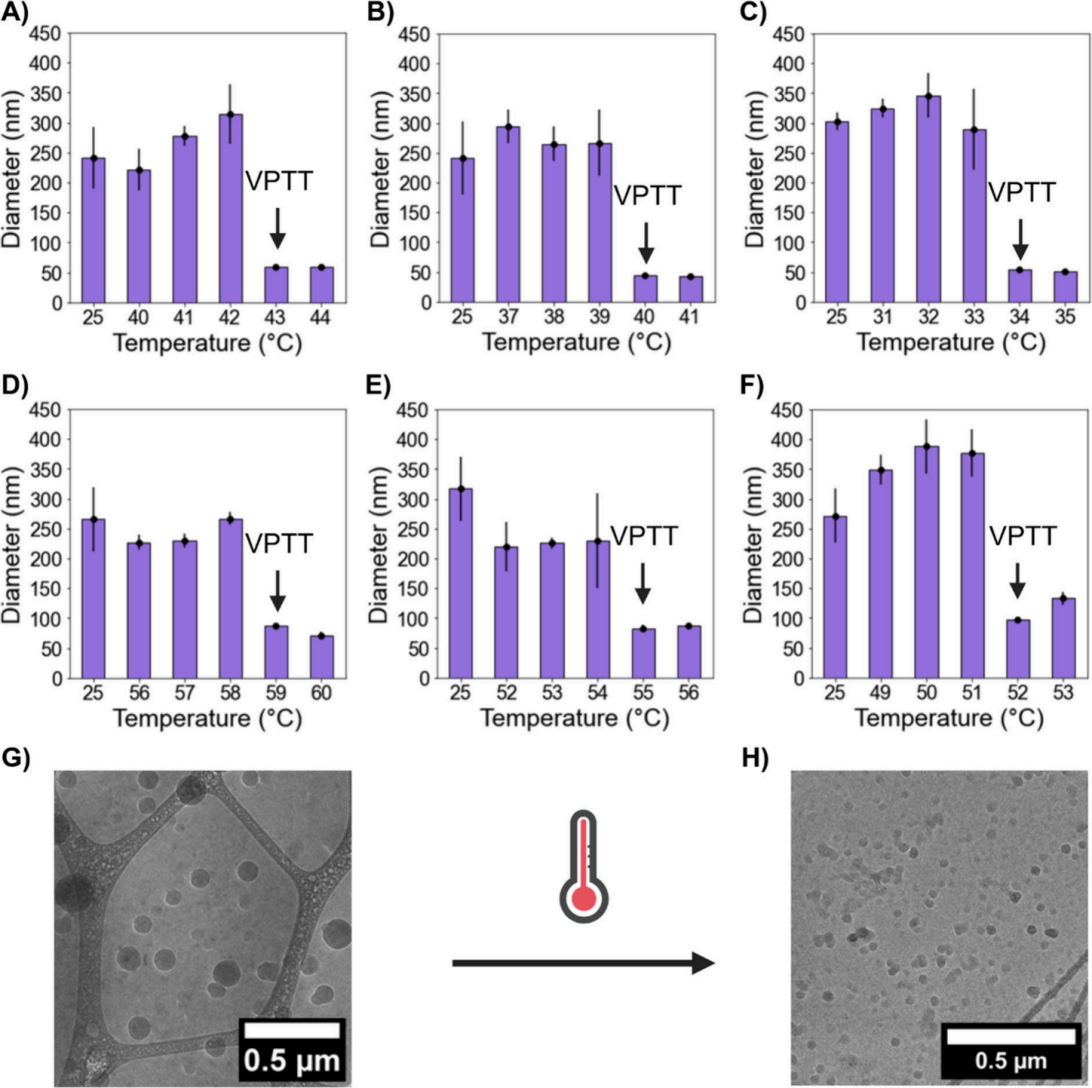
Characterization of exact volume phase transition temperatures (VPTTs) (hydrodynamic diameter vs temperature measurements) in water for (A) NG@DEGMA_1, (B) NG@DEGMA_2, (C) NG@DEGMA_3, (D) NG@TEGMA_1, (E) NG@TEGMA_2, and (F) NG@TEGMA_3. (G) Representative cryo-TEM image of NG@TEGMA_3 prepared at 4 °C, magnification 38 kx, scale bar 0.5 μm. (H) Representative cryo-TEM image of NG@TEGMA_3 prepared at 60 °C, magnification 38 kx, scale bar 0.5 μm. All images were acquired with accelerating voltage (AV) 200 kV. In all cases, data are presented as mean ± standard deviation for *n* = 3.

UV–vis transmittance measurements were performed on a representative formulation (NG@TEGMA_3). While a discrete thermoresponsive transition was observed starting at 58 °C, Figure S13, the transmittance decreased only to approximately 60% before recovering at higher temperatures. This optical profile, combined with the small size of NGs after the transition, Table S2, confirmed that the NGs undergo an individual structural collapse rather than macroscopic aggregation or phase separation.^37^ Because these NGs behave as stable, discrete colloids rather than a phase-separating bulk polymer above VPTT, traditional turbidimetry, which relies on the formation of a turbid heterogeneous phase, was found to be less sensitive than DLS.^38^ Consequently, DLS was utilized as the primary characterization tool for all formulations, as it provides a more precise and direct quantification of the hydrodynamic volume change that defines the VPTT in these monodisperse systems.^37^

To achieve a VPTT of around 40 °C within 1 °C, the combination of two monomers was investigated. The NG@TEGMA_DEGMA showed a VPTT above 60 °C, Figure 5A–C. While DEGMA has a lower intrinsic VPTT, the higher hydrophilicity of TEGMA dominated the polymer–water interactions, stabilizing the swollen state. Consequently, the NG required a higher temperature to collapse, resulting in a VPTT similar to that of pure TEGMA. This highlights that mixing two hydrophilic monomers is insufficient to reduce the VPTT, and the introduction of a hydrophobic comonomer is necessary to achieve lower transition temperatures.^39^ Interestingly, NG@TEGMA_DEGMA_3 appeared less monodispersed under cryo-TEM, Figure 5D, than NG@TEGMA_3, Figure 4G.

**Figure 5:**
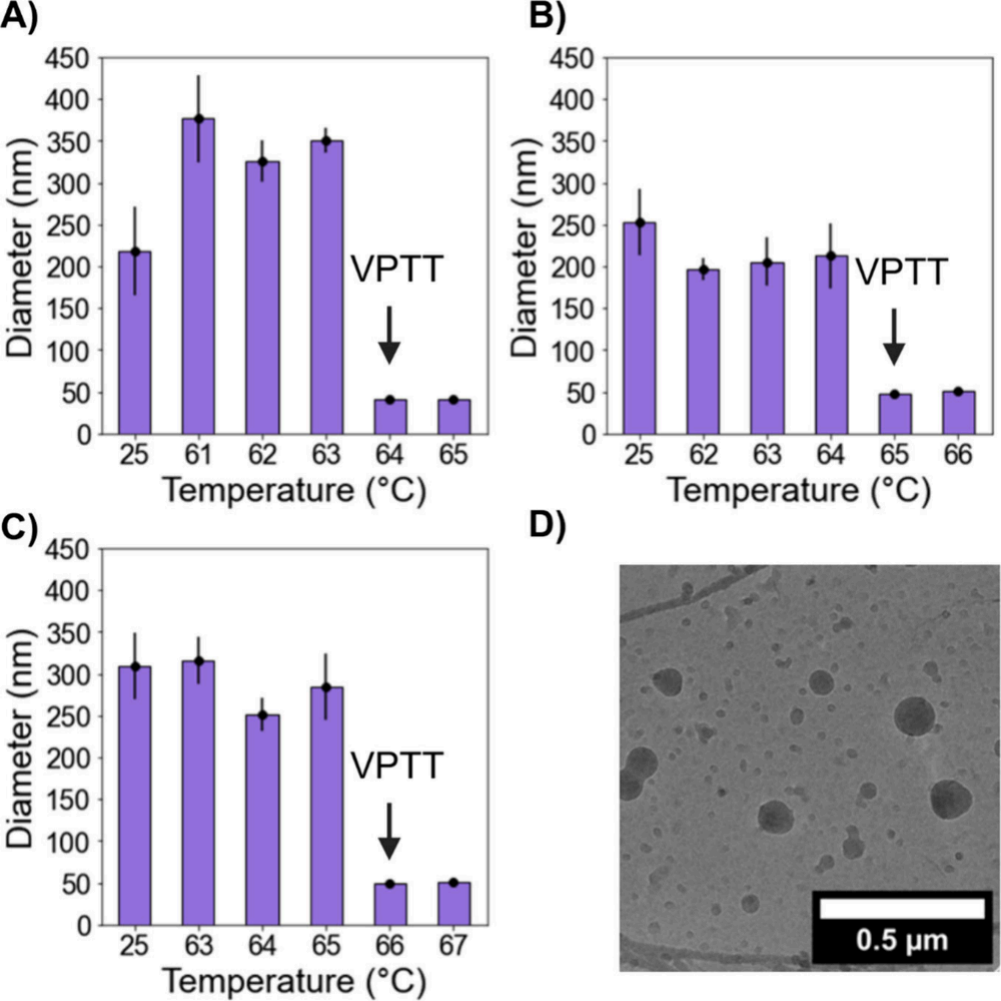
Characterization of exact volume phase transition temperatures (VPTTs) (hydrodynamic diameter vs temperature measurements) in water for (A) NG@TEGMA_DEGMA_1, (B) NG@TEGMA_DEGMA_2, and (C) NG@TEGMA_DEGMA_3. (D) Representative cryo-TEM image of NG@TEGMA_DEGMA_3 prepared at 4 °C, magnification 38 kx, scale bar 0.5 μm. All images were acquired with accelerating voltage (AV) 200 kV. In all cases, data are presented as mean ± standard deviation for *n* = 3.

To lower the VPTT, MOEMA was investigated as a hydrophobic comonomer. It is well established in the literature that incorporating a hydrophobic comonomer reduces the LCST of bulk polymers and the VPTT of NGs.^26^ NG@MOEMA showed a much smaller diameter than the other formulations (Figure 6E) and had no VPTT: Figure 6D shows that the diameter of NG@MOEMA remained unchanged as the temperature rose.^29^ However, NGs composed of TEGMA and MOEMA showed a sharp decrease in VPTT. The following TEGMA to MOEMA molar ratios were investigated: 80:20, 70:30, and 60:40, resulting in VPTT of 52, 47, and 37 °C, respectively, Figure 6A–C. In fact, incorporation of the hydrophobic monomer MOEMA into TEGMA-based NGs reduced polymer–water interactions and destabilized the hydrated polymer network.^20^ This promoted an earlier collapse of the NG, resulting in a lower VPTT. This demonstrated that by carefully selecting the ratio of TEGMA to MOEMA, it was possible to achieve a wide range of transition temperatures.

**Figure 6:**
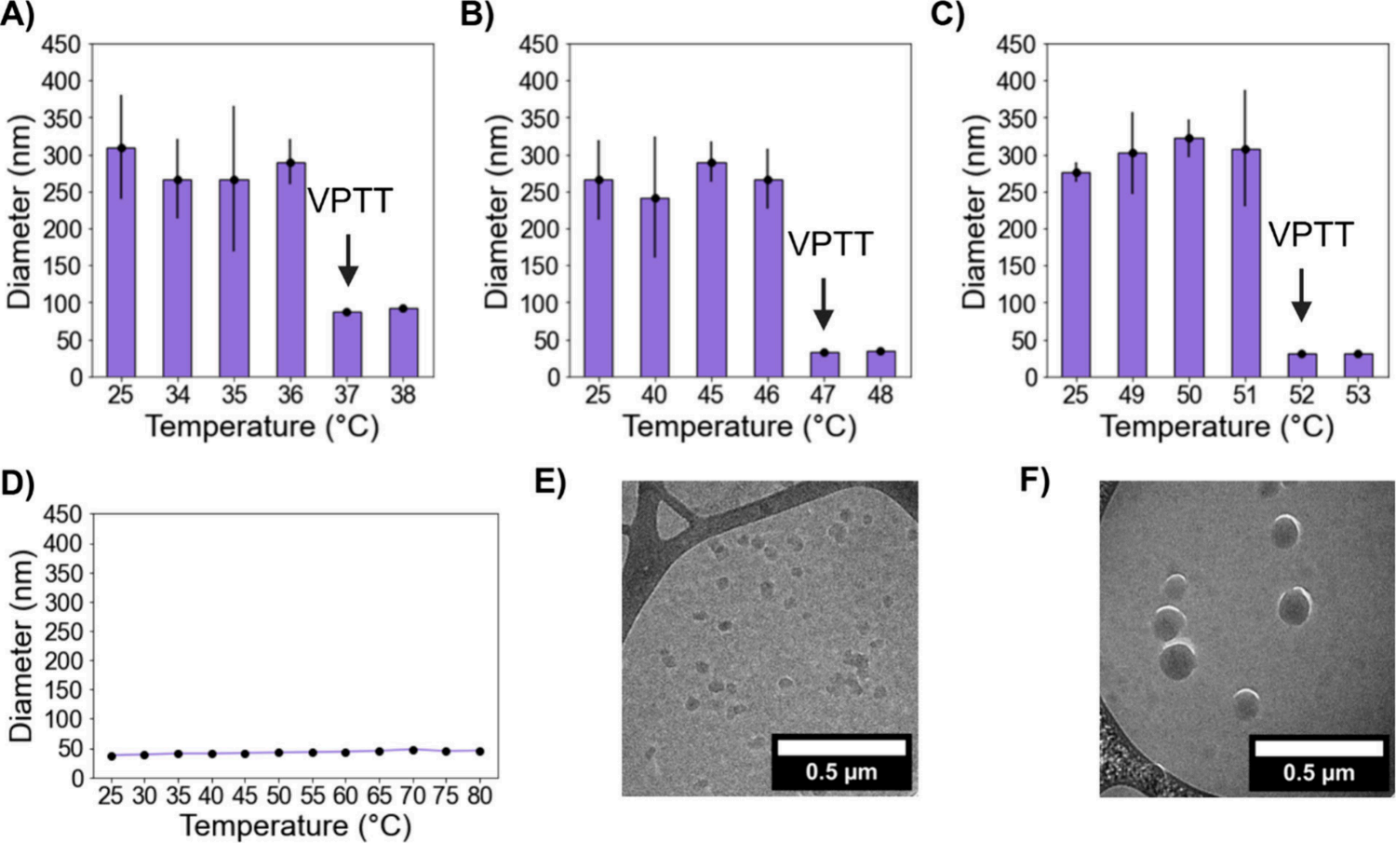
Characterization of exact volume phase transition temperatures (VPTTs) (hydrodynamic diameter vs temperature measurements) in water for (A) NG@TEGMA_MOEMA_1, (B) NG@TEGMA_MOEMA_2, and (C) NG@TEGMA_MOEMA_3. (D) Size vs temperature measurements obtained with DLS for NG@MOEMA showing no VPTT. (E) Representative cryo-TEM image of NG@MOEMA prepared at 4 °C, magnification 38 kx, scale bar 0.5 μm. (F) Representative cryo-TEM image of NG@TEGMA_MOEMA_3 prepared at 4 °C, magnification 38 kx, scale bar 0.5 μm. All images were acquired with accelerating voltage (AV) 200 kV. In all cases, data are presented as mean ± standard deviation for *n* = 3.

The diverse and sharp transition temperatures of the NGs are governed by a synergistic interplay between the polymer’s hydrophilic/hydrophobic balance and the network’s structural homogeneity. The relationship between the monomer molar amount and the VPTT is illustrated through a linear regression analysis of the NG@DEGMA, NG@TEGMA, and NG@MOEMA series, Figure S14. The analysis reveals a clear, quasi-linear dependence which suggests that the VPTT can be precisely tuned by adjusting the molar quantity of the specific OEGMA building blocks. As shown by the negative slopes, the incorporation of these monomers results in a predictable decrease in VPTT;^20,28,26^ for every 0.001 mmol of DEGMA added to the feed, the VPTT decreases by approximately 1 °C, while for every 0.001 mmol of MOEMA added, the VPTT drops by nearly 3 °C. Due to the higher hydrophobic nature of MOEMA (possessing only one EG chain), the decrease in VPTT is sharper.^20,28^ Similarly, the TEGMA series displays a negative linear correlation; while typically characterized as a hydrophilic monomer,^21,36^ the higher hydrophilic nature of the control network causes TEGMA to act as a hydrophobic moiety that lowers the VPTT by 0.7 °C for every 0.001 mmol added, albeit less strongly than DEGMA or MOEMA. Interestingly, the NG@TEGMA_DEGMA series maintains a high and relatively stable VPTT (64–66 °C), suggesting that the transition does not always follow a simple linear hydrophilic–hydrophobic balance.^36^ In fact, it has been observed that the transition of a copolymer with a hydrophobic comonomer and a hydrophilic comonomer can be complicated by intrachain or interchain self-assembly.^36,40^

Significantly, all NG formulations exhibited a sharp size reduction within 1 °C range at the VPTT. Most of the thermoresponsive OEGMA-based NGs reported in the literature display broader and less defined VPTT, with size reductions occurring gradually over several degrees (°C).^24,25,27,31^ Furthermore, the extent of size reduction in previously reported systems is typically less pronounced. For example, Macchione et al. reported a decrease in diameter between 390 and 330 nm for heating between 25 and 75 °C.^27^ Similarly, the NGs synthesized by Zhang et al. showed a decrease from 320 to 200 nm between 25 and 45 °C, with minimal change in PDI (0.239 and 0.289 at 25 and 45 °C, respectively).^25^ Other thermoresponsive systems, such as NIPAM- and VCL-based NGs, exhibit similarly broad transitions and less substantial size changes.^15,31^ In contrast, the NGs synthesized in this study displayed a sharp and substantial decrease in size to below 100 nm at the VPTT, Table S2, demonstrating enhanced and well-defined thermoresponsive behavior. The exceptionally sharp VPTT (ΔT = 1 °C) observed is a direct consequence of the optimized network architecture and high segment mobility.^41^ The Flory–Rehner equation, eq S1, was used to calculate the average molecular weight between cross-link (*M*_*c*_) and determine the level of cross-linking. The structural parameters obtained from the equation, specifically the high *M*_*c*_ (10^4^–10^7^ g/mol),^42^ the low polymer volume fractions (*φ*_*2*_, eq S2) (<0.05)^43^ and high linear swelling ratios (*S*_*D*_, eq S3) (mostly above 3.5),^44,45^ suggested low cross-linking density and formation of ultralow cross-linked or ultrasoft NGs with high water content,^45^ Table S3. These water-rich, expanded matrices experienced an abrupt phase transition due to a lower elasticity component and reduced steric hindrance.^41,43,46^ Hence, the dehydration of the EG side chains triggered a near-instantaneous collapse of the network, which provided the sharp transition observed across in these NGs.

For all the formulations, the VPTT also influenced the PDI of the NGs’ solutions. Immediately after synthesis, the PDI was high, reflecting a polydisperse population, Table S1. Upon reaching the VPTT, a marked decrease in PDI was observed, indicating that the NGs adopt a monodisperse, collapsed state, as summarized in Table S2. In contrast, OEGMA-, NIPAM-, and dextran-methacrylate- NGs reported in the literature frequently undergo severe aggregation above their VPTT, reflected in large particle sizes and elevated PDIs.^15,24,26,31^ Such aggregation may adversely affect their behavior in biological environments.^47^ Overall, the OEGMA-based NGs developed in this work exhibit improved thermoresponsive sharpness and reduced aggregation, which makes them promising candidates for drug delivery applications.

A cyclic DLS experiment was performed to show that the size reduction is not caused by polymer degradation and that the thermoresponsive behavior of the NGs was reversible, as is typically observed for OEGMA-based systems.^20^ Briefly, the NGs were repeatedly heated above VPTT and subsequently cooled to room temperature, with an equilibration time of 1 h at each temperature point. Figure 7 shows that the NGs retain their VPTT even after multiple heating and cooling cycles. Notably, after the heating above their VPTT, the NGs returned to their original size upon cooling to 25 °C, demonstrating fully reversible behavior. This shrink–swelling process could be repeated up to 5 times, while previously reported OEGMAs NGs could only reach up to three cycles.^26^ The recovering of the diameter after cooling the solution indicated that the NG matrix remains structurally intact at elevated temperatures and that the observed DLS size changes result solely from conformational transitions within the polymer network.^37^ This cycle study served as a mechanistic confirmation of the network’s reversibility, consistent with protocols established for other NGs systems.

**Figure 7:**
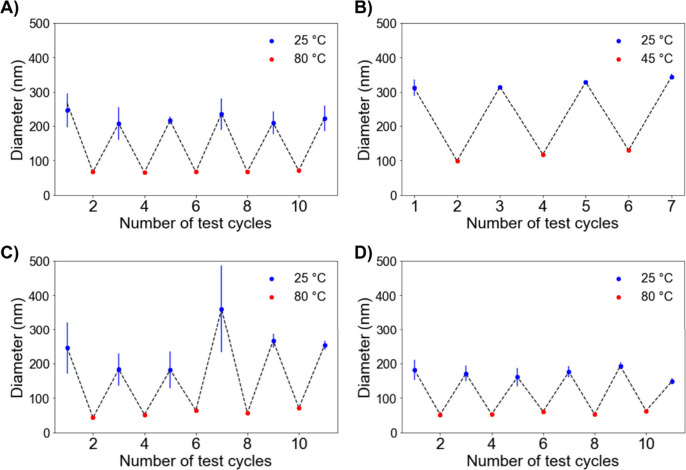
Cyclic measurements in water for (A) NG@TEGMA_3, (B) NG@DEGMA_2, (C) NG@TEGMA_DEGMA_2, and (D) NG@TEGMA_MOEMA_3. Equilibration time is 1 h. In all cases, data are presented as mean ± standard deviation for *n* = 3.

Finally, little to no change in ζ-potential was observed upon reaching the VPTT, Table S2, with all NG formulations maintaining a negative surface charge, ranging from −14 mV for NG@DEGMA_3 to −36 mV for NG@TEGMA_1. This behavior is consistent with the low PDIs measured above the VPTT, indicating that the NGs remain well dispersed in solution despite their reduced size.

For subsequent studies, including stability, drug encapsulation and release, and biological characterization, NG@TEGMA_3 (from here on called NG@TEGMA) was selected as the model NG due to its well-defined LCST, greater monodispersity, and response at elevated temperatures.

### Stability and Degradation Studies

To determine the suitability of the thermoresponsive NGs for biomedical applications, we monitored changes in their properties during long-term storage and at physiological conditions. First, NG@TEGMA was stored at 4 °C after synthesis, and its size, PDI, and ζ-potential were monitored over 100 d at predetermined time points, Figure S15A, C, E. The particle size remained largely unchanged over this period, with some swelling observed after 60 d; however, the PDI oscillated between 0.3 and 0.6. The ζ- potential increased from −25 to −15 mV, which could promote some aggregation and contribute to variations in PDI.

Next, NG@TEGMA solutions in DI water and PBS were incubated at 37 °C, and their size, PDI, and ζ-potential were monitored over 1 week, Figure S15B, D, F. Under these conditions, degradation of the NGs due to ester hydrolysis was anticipated.^41^ Both solutions remained stable until 2 d, suggesting bulk degradation of the polymer matrix, which does not cause a size change.^48^ From 3 d onward, swelling and aggregation were observed, indicated by rapid changes in size and PDI and by shifts in ζ-potential.^30^ TEM images confirmed the degradation of the NGs between 3 and 7 d, Figure S16.

To further evaluate the colloidal stability of the NGs under clinically relevant conditions, NG@Control and NG@TEGMA were incubated in DI water, DMEM (cell culture media), and DMEM supplemented with 10% Fetal Bovine Serum (FBS) at 37 °C for 2 h. As shown in Figure S17A and B, the hydrodynamic diameter of both NG formulations remained stable across all media, with no significant increase in size observed upon exposure to serum proteins. While an increase in the PDI was noted in the group exposed to DMEM + FBS, Figure S17C, D, this was attributed to the intrinsic light-scattering contribution of bulky serum proteins (e.g., bovine serum albumin) rather than NG aggregation. The maintenance of the primary particle size confirms that the EG brushes of the NGs provided steric stabilization and resistance to nonspecific protein adsorption.^20^ These results, combined with the 48-h stability observed in PBS experiments, Figure S15, demonstrated that the NGs possess the necessary robustness for operation in physiological environments.

### Characterization of VPTT in DMEM

The VPTT of the NG@TEGMA series was investigated in DMEM supplemented with 10% FBS, Table S4. Due to the high-ionic-strength environment and the presence of biomacromolecules, the NGs exhibited lower VPTT and aggregation after the transition, with diameters increasing from around 300 nm to around 1200 nm. This reduction of the VPTT is a well-documented characteristic of OEGMA-based polymers and NIPAM NGs in the presence of physiological salts^49,50^ and proteins.^51,52^ The electrolytes induce a salting-out effect that modulates the VPTT without compromising the fundamental thermo-reversibility of the system. Furthermore, the increase in size and the aggregation were attributed to the formation of a protein corona, driven by the higher hydrophobicity of the NGs’ surface after VPTT.^52,53^ The thermoresponsiveness was further investigated over 5 consecutive heating/cooling cycles between 25 and 80 °C, Figure S18. Consistent with the experiments carried out in water, these data confirmed that the NGs’ shrinking/swelling process is also reversible in biological media. However, a gradual downward trend in the aggregate diameter and a slight elevation of the baseline size were observed over successive cycles; this drift is likely attributed to the formation of the protein corona, with this behavior previously observed for flexible NGs with low cross-linking density.^51^ The cyclic study serves as a mechanistic confirmation of the network’s stability even in complex media. Collectively, these results demonstrate that the TEGMA-based architecture maintains robust and reversible thermoresponsive behavior at temperatures appropriate for hyperthermia or ultrasound therapy, even in biological environments.

### Drug Encapsulation

One of the major applications of thermoresponsive polymers is for stimuli-responsive drug delivery systems, which can be employed in various therapeutic contexts, including chemotherapy. As a proof of concept, NG@TEGMA was selected to demonstrate the controlled delivery of the chemotherapy drug docetaxel (DTX).

For sustained release, DTX was functionalized with mono-2-(methacryloyloxy)­ethyl succinate via an ester coupling to yield **1**, Figure 8A. Details of the synthetic procedure are provided in the experimental methods. While esterification of DTX’s hydroxyl groups has been reported previously,^54–56^ **1** represents, to our knowledge, the first example of a methacrylate-functionalized DTX. The methacrylate group enables copolymerization with the NG matrix, thereby covalently incorporating the drug and preventing premature leakage. The formation of the compound was confirmed via ^1^H NMR and MS, Figures S8 and S11. In the ^1^H NMR, the characteristic vinyl proton signals of the methacrylate group are observed, which were absent in the spectrum of unmodified DTX. Furthermore, a downfield shift of the H_j_ resonance after esterification provided additional confirmation, Figures S9 and S10.

**Figure 8:**
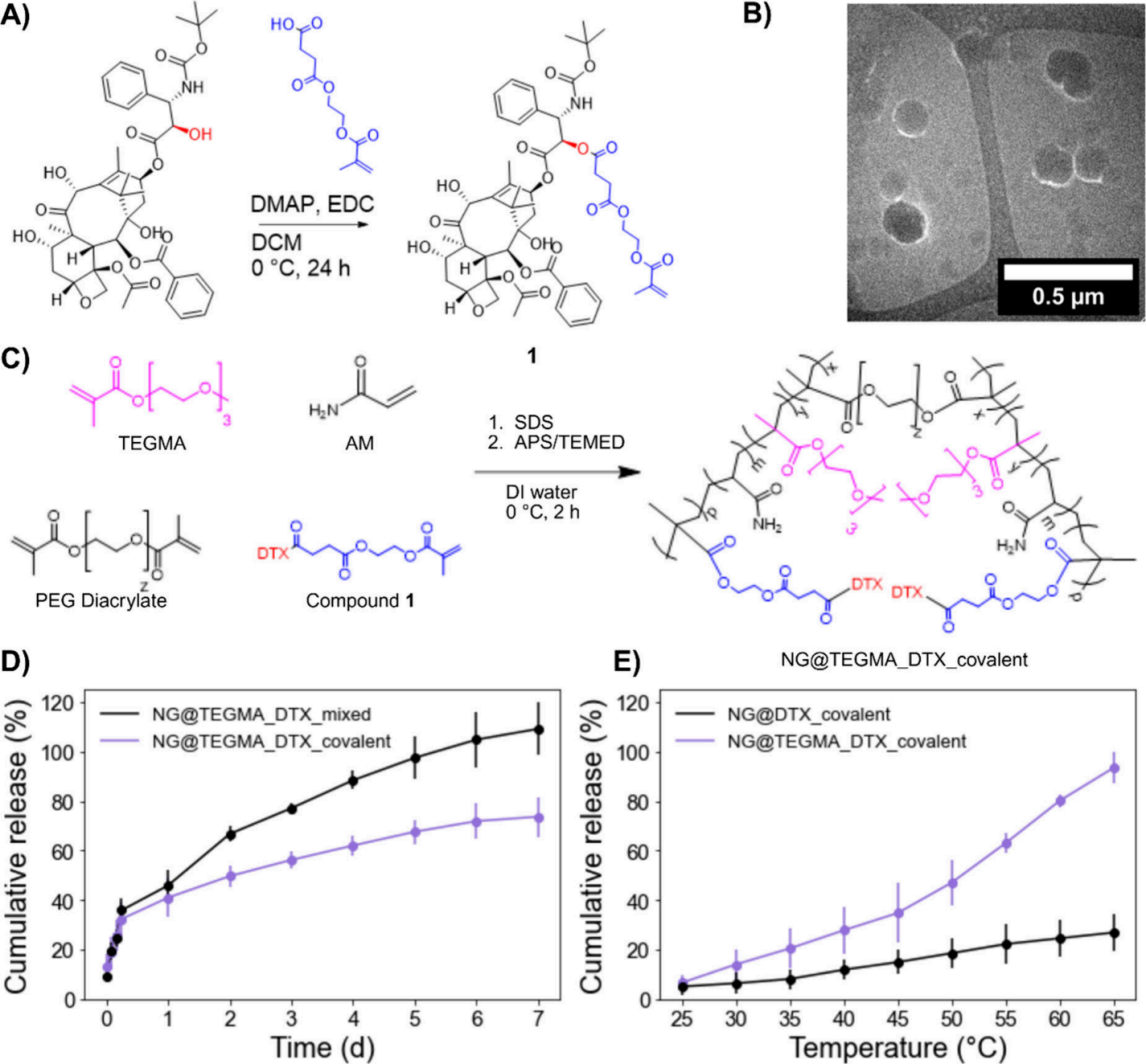
(A) Synthesis scheme for **1**. Abbreviations: DMAP = 4-(dimethylamino)­pyridine, EDC = 1-ethyl-3-(3-(dimethylamino)­propyl)­carbodiimide, DCM = dichloromethane. (B) Representative cryo-TEM image of NG@TEGMA_DTX_covalent, magnification 38 kx, scale bar 0.5 μm, acquired with accelerating voltage 200 kV. (C) Synthesis scheme for NG@TEGMA_DTX_covalent in distilled (DI) water. Abbreviations: TEGMA = tri­(ethylene glycol) methyl ether methacrylate, AM = acrylamide, PEG diacrylate = polyethylene glycol diacrylate 250 MW, DTX = docetaxel, SDS = sodium dodecyl sulfate, APS = ammonium persulfate, TEMED = *N,N,N′,N′*-tetramethylethylenediamine. (D) Cumulative release from NG@TEGMA_DTX_covalent and NG@TEGMA_DTX_mixed incubated at 37 °C for 7 d. (E) Cumulative release from NG@TEGMA_DTX_covalent and NG@DTX_covalent with rising solution temperature. In all cases, data are presented as mean ± standard deviation for *n* = 3.

### Drug Loading

NG@TEGMA_DTX_covalent was synthesized following the general NG protocol as the methacrylate group of **1** (highlighted in blue in Figure 8A) allows for the DTX to be polymerized in the NG matrix along with the other monomers. Cryo-TEM images of the NGs are shown in Figure 8B and NG synthesis scheme in Figure 8C. The resulting NGs exhibited a hydrodynamic diameter of 291 ± 28 nm and a ζ-potential of −11 mV, Table S5. Following the incorporation of DTX, the ζ-potential of NG@TEGMA_DTX_covalent increased relative to that of NG@TEGMA (−25 mV). Nevertheless, NG@TEGMA_DTX_covalent retained a sufficiently negative surface charge to ensure colloidal stability and facilitate cellular uptake, consistent with previous reports.^8^ Importantly, NG@TEGMA_DTX_covalent preserved the thermoresponsive behavior of the parent NG@TEGMA system, exhibiting a sharp size reduction and an improved PDI at the VPTT, as summarized in Table S6.

Both the encapsulation efficiency (EE%) and the loading efficiency (LE%), Table S5, were higher than other DTX loaded systems such as poly­(D,l-lactide-*co*-glycolide)/hyaluronic acid nanoparticles (87% and 2.71%)^57^ or micelles (85% and 7.5%).^58^ In addition, the EE and the LE were slightly higher than those reported for other NGs systems where DTX was physically entrapped.^59,60^ In these NGs, the EE% was found to be around 60–90% and the LE% 10–15%. For drug release studies, a control formulation NG@TEGMA_DTX_mixed was prepared, in which unmodified DTX was physically entrapped within the NG matrix without covalent incorporation.

Both NG@TEGMA_DTX_covalent and NG@TEGMA_DTX_mixed were incubated in a water bath at 37 °C, and release profiles were monitored over 7 d (Figure 8D). NG@TEGMA_DTX_covalent exhibited markedly improved control over DTX release compared to the physically loaded control, NG@TEGMA_DTX_mixed. In NG@TEGMA_DTX_covalent, DTX was covalently conjugated to the polymer matrix via an ester bond (highlighted in red in Figure 8A), which is hydrolytically labile under physiological conditions.^61^ Over the 7-d period, NG@TEGMA_DTX_covalent released approximately 50% of DTX at 2 d, 60% at 4 d and 70% at 7 d, whereas the physically encapsulated NG@TEGMA_DTX_mixed released around 70% of the drug within just 2 d. By covalently incorporating DTX, the NG exhibited a slower, more controlled release profile compared with physical encapsulation, thereby overcoming the common issue of premature drug leakage. This behavior is well characterized in polymer–drug conjugates for drug delivery.^55^

Our NGs system compares favorably with previously reported DTX delivery systems (both physically entrapped and covalently conjugated), offering enhanced temporal control and sustained release. For example, Huang et al. developed poly­(D,l-lactide-*co*-glycolide)/hyaluronic acid NGs with noncovalently encapsulated DTX (DTX/SANPs) containing noncovalently encapsulated DTX, which released approximately 60% of the drug within 20 h.^57^ Similarly, the PEG–PCL–DTX polymer–drug conjugate micelles by Mikhail et al. released nearly 50% of DTX within 1 d.^55^ In contrast, NG@TEGMA_DTX_covalent and NG@TEGMA_DTX_mixed released only 40% of the drug at the same time point. These findings established a clear proof-of-concept that covalent conjugation within a thermoresponsive NG matrix enables fine modulation of drug release kinetics, providing a promising strategy for sustained and heat triggered chemotherapy delivery.

To assess thermoresponsive release behavior, NG@TEGMA_DTX_covalent was gradually heated from 25 to 65 °C, with samples collected at 5 °C intervals and equilibration time of 2 min (with total experiment time of around 15 min). As shown in Figure 8E, approximately 90% of the drug was released after reaching the VPTT (52 °C) for NG@TEGMA_DTX_covalent, compared with only 20% release from the nonthermoresponsive control NG@DTX_covalent. This demonstrates that the VPTT of the NG facilitates triggered drug release under thermal stimulation. The release profile of NG@TEGMA_DTX_covalent aligns with those reported for other thermoresponsive nanoparticle systems. For instance, Kono et al. observed temperature-dependent release in NIPAM-based liposomes, with 20% calcein released below the VPTT (35 °C) and nearly complete release above it in approximately 2 min.^62^ Similarly, NIPAM-based thermosensitive liposomes with physically entrapped doxorubicin (DOX) exhibited approximately 10% and up to 65–90% release before and after the VPTT, respectively.^63,64^ However, as mentioned previously, NIPAM-based materials are known to have limited clinical applicability due to cytotoxicity^11,13,14^ and a relatively low VPTT that restricts therapeutic flexibility. In contrast, the NG@TEGMA_DTX_covalent system offers a fully biocompatible and tunable alternative capable of operating within the optimal temperature window for high temperature-driven drug delivery.

Liquid chromatography–mass spectrometry (LC-MS) was employed to evaluate ester-cleavage-mediated release of DTX from the NG conjugate following release experiments, Figures S19 to S22. Analysis of NG@TEGMA_DTX_covalent at 25 °C confirmed the presence of intact compound **1**, with detection of the expected conjugate molecular ions, demonstrating that compound **1** was initially present in a chemically bound form, Figure S20. After both short-term heating at 60 °C (15 min) and long-term incubation at 37 °C (3 d), these conjugate ions were no longer observed, indicating chemical cleavage of the ester linker under release conditions, Figure S20 and S21. The ester linkage is unlikely to have been cleaved during the mass spectrometric analysis itself, as intact conjugate molecular ions were observed in NG@TEGMA_DTX_covalent, demonstrating that the ester bond is stable in the ion source and that cleavage occurs prior to MS detection. The release samples, instead, exhibited mass spectral features consistent with DTX, which matched those obtained from a DTX reference standard analyzed under identical chromatographic and ionization conditions, Figure S19. These included DTX molecular ions as well as the appearance of well-established diagnostic fragment ions, most notably the phenylisoserine side-chain ion around *m*/*z* 265 together with taxane-core fragments (e.g., *m*/*z* approximately 387–413).^65^ Although intact DTX molecular ions were less prominent in the release samples than in the concentrated reference standard, this is attributed to the substantially lower analyte concentration and increased matrix complexity of the release media, both of which are known to enhance in-source fragmentation and suppress parent-ion abundance in ESI.^66^ Importantly, no additional mass spectral features indicative of alternative degradation products was detected. Collectively, these data demonstrated conversion of the conjugate and release of structurally intact DTX, consistent with liberation of the pharmacologically active drug via ester cleavage.

### Cell Viabilities

To assess the potential cytotoxicity of NG@TEGMA, NG@TEGMA_DTX_mixed and NG@TEGMA_DTX_covalent, HeLa cancer cells were incubated with the NGs for 24 h. The relative cell viabilities at different NG concentrations were evaluated using the CCK-8 assay, Figure 9A. NG@TEGMA showed negligible impact on cell viability across the tested concentration range. NG@TEGMA_DTX_covalent did not induce marked cytotoxicity up to 150 μg/mL, suggesting that the covalently incorporated drug remains largely shielded within the NG matrix but starts to show a slight cytotoxic effect at higher concentrations. On the other hand, the NGs that contained the drug which was not covalently bonded (NG@TEGMA_DTX_mixed), showed high cytotoxicity. Hence, the drug incorporation reduces premature exposure of cells to DTX. This shielding effect is particularly advantageous in reducing off-target toxicity, a known limitation of conventional DTX administration.^67,68^ These findings correlated with the controlled release profile observed for NG@TEGMA_DTX_covalent, where DTX release occurred gradually through ester bond hydrolysis. Together, these results demonstrated that the NG matrix both protected against premature cytotoxicity and enabled control of drug release. Overall, NG@TEGMA maintained cell viabilities above 80% at concentrations up to 250 μg/mL, indicating low inherent toxicity of the polymer platform. Notably, this cyto-compatibility compares favorably to other thermoresponsive NGs reported in the literature,^25–27^ even in the presence of encapsulated DTX.

**Figure 9:**
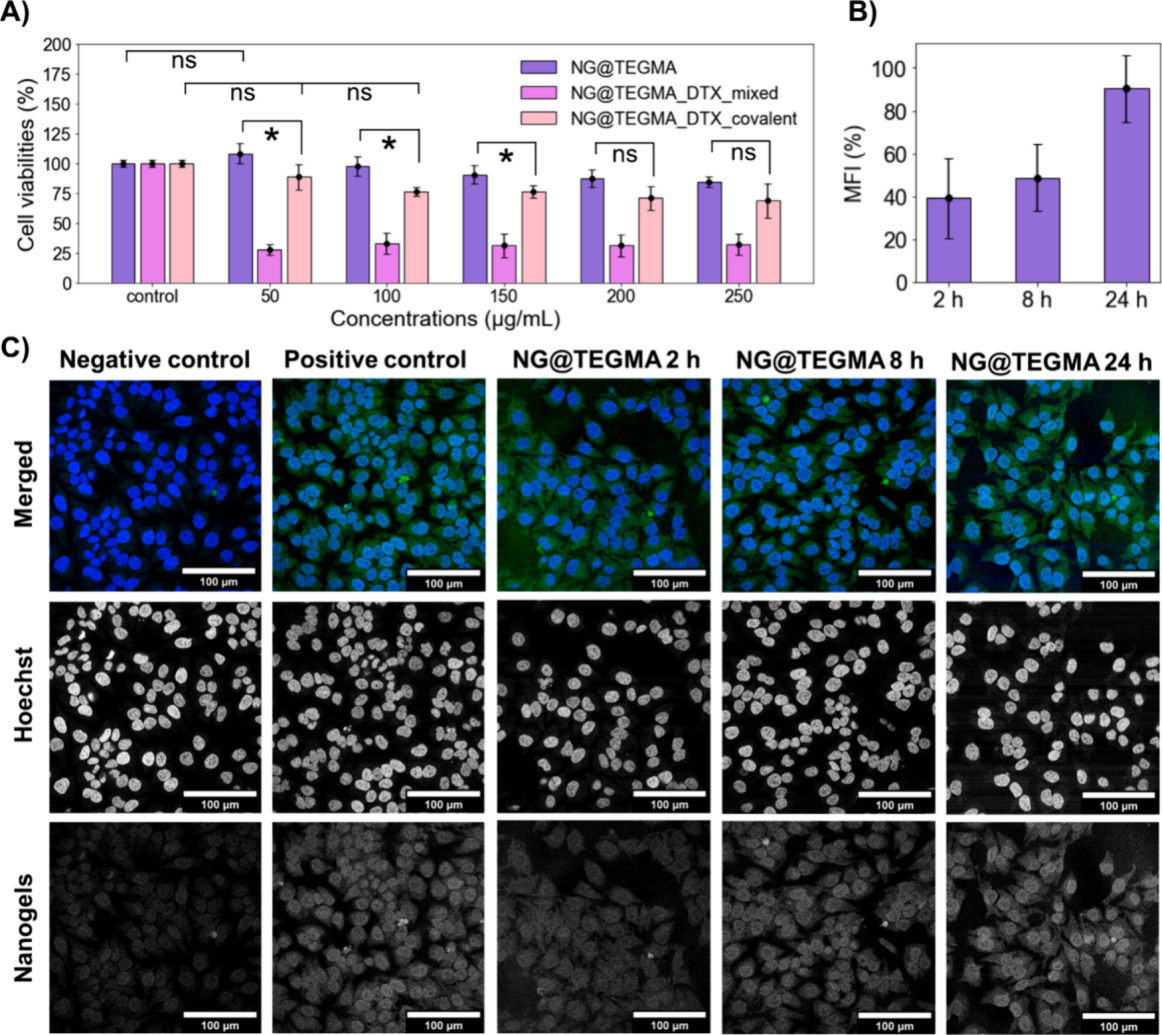
(A) Cell viabilities of HeLa cells (%, *n* = 3) assessed by CCK-8 assay after exposure to different concentrations of NG@TEGMA, NG@TEGMA_DTX_mixed, NG@TEGMA_DTX_covalent for 24 h. For NG@TEGMA_DTX_mixed and NG@TEGMA_DTX_covalent, the DTX concentration is 3.2 and 9.3 wt %/wt of the formulation, respectively. The control group represents cells with no treatment. Data shown are mean ± SD of biological replicates. (B) Quantification of intracellular uptake of NG@TEGMA compared to positive control by ImageJ quantitation method.^69^ Mean fluorescence intensities (MFI%) of the different time points are shown. Data shown are mean ± SD of *n* = 15. (C) Confocal microscopy images showing the intracellular uptake of fluorescein-labeled control NGs and NG@TEGMA in HeLa cells, fixed 2, 8, 24 h post-treatment, stained with 1 μg/mL Hoechst 33342. Images were taken with 40x oil immersion objective. Scale bar at 100 μm. Statistical significance was determined using an unpaired *t* test, *p < 0.05, **p < 0.005, and ns >0.05;.

### Cellular Uptake

The cellular uptake of fluorescein-labeled NG@TEGMA by HeLa cells was evaluated using confocal laser scanning microscopy. Representative images are shown in Figure 9C, alongside positive control.^5^ In the merged channel, nuclei are stained blue, while fluorescein-labeled NGs appear green. The NGs were clearly internalized by the cells and localized within the cytoplasm as early as 2 h postincubation, with maximal accumulation observed after 24 h. These results confirmed the ability of NG@TEGMA to penetrate cancer cells, supporting their potential application as drug delivery vehicles.

## Conclusions

In this study, a series of thermoresponsive OEGMA-based NGs were synthesized and systematically engineered to achieve finely tunable VPTT within physiologically and therapeutically relevant temperature ranges. The NGs demonstrated superior thermoresponsive behavior to previously reported OEGMA-, NIPAM- or VCL-based NGs, exhibiting a sharp and reversible volume phase transition with minimal aggregation and enhanced monodispersity. They also maintained structural stability during storage, showing their robustness. Additionally, they exhibited significant structural changes within 2 d of physiological incubation, suggesting susceptibility to biodegradation and potential for efficient clearance. NG@TEGMA_DTX_covalent, with covalently incorporated DTX, demonstrated high encapsulation efficiency and sustained drug release, with accelerated release near the VPTT. NG@TEGMA was noncytotoxic and NG@TEGMA_DTX_covalent was well-tolerated up to around 150 μg/mL, with the expected cytotoxicity emerging at higher doses as DTX is released. The formulation also showed efficient cellular internalization, highlighting their biocompatibility.

Overall, the ability to adjust the transition temperature with single-degree precision provides a versatile strategy for designing NGs tailored to specific biomedical conditions, including hyperthermia-assisted chemotherapy and ultrasound focal therapy, establishing these NGs as a promising next-generation carrier for controlled and targeted drug delivery.

## Experimental Methods

### Materials

Docetaxel was purchased from Molekula Limited, UK. All other reagents were purchased from Sigma-Aldrich, UK and used as received without any further purification. Anhydrous ethyl acetate (EtOAc) and methanol (MeOH) were used as supplied, with anhydrous dichloromethane (DCM) acquired by filtration through drying column towers. Amicon Ultra 0.5 mL (100 kDa Molecular Weight Cut-Off, MWCO) and 50 mL (30 and 100 kDa MWCO) Centrifugal Filter Units were purchased from Sigma-Aldrich, UK. 1.5 mL Eppendorf Safe-Lock Tubes were purchased from Fischer Scientific, UK. DI Water used was purified using a Milli-Qpurification system and the sterile 0.25 filters were purchased from Pall Corporation. 40% acrylamide in water solution was used for the synthesis. HeLa cells were obtained from American Type Culture Collection (ATCC), UK. GibcoTM Dulbecco’s Modified Eagle’s Medium (DMEM) was purchased from Thermo Scientific Chemicals, UK. The Cell Counting Kit-8 (CCK-8) was purchased from Stratech Scientific, UK.

### Instrumentation

^1^H were recorded on a Bruker AV-400 NMR spectrometer. Chemical shifts are expressed in parts per million (ppm) and are referenced to the residual solvent peak (δ_H_ 7.26, CDCl_3_). In the assignment of the NMR peaks, letters are given in the brackets, which correspond to the proton labeling in the relevant structure. Coupling constants (*J*) are measured in Hz with the following abbreviations: *s* = singlet, *d* = doublet, *t* = triplet, *q* = quartet, *m* = multiplet. Blanks of NMR solvent only (CDCl_3_) were run to confirm the solvent purity. Dynamic light scattering (DLS) and electrophoretic light scattering (ELS) analysis was performed on a Malvern Zetasizer Ultra instrument. The UV–vis transmittance measurements were done using a Cary 3500 Compact Peltier UV–Vis System (Agilent). Negative stained (NS) Transmission Electron Microscopy (TEM) images were obtained using JEOL 2100Plus TEM, with an electron accelerating voltage (AV) of 200 kV. Cryo-TEM images were obtained using a Tecnai T20 G2, with an AV of 200 kV. Absorbance for cell viability assays was measured with a SpectraMaxABS Plus Microplate Reader at 450 nm. Thin layer chromatography (TLC) plates were visualized using a SpectrolineENF 260C hand-held UV lamp. 1290 Infinity III UHPLC DAD/FLD/ELSD (Agilent Technologies, Santa Clara, CA, USA) was used for chromatographic analysis (HPLC) equipped with ZORBAX RR Eclipse XDB-C8 Column (Agilent Technologies, Santa Clara, CA, USA, 4.6 mm × 75 mm, 3.5 μm). Fourier transform infrared spectra (FT-IR) was collected using Agilent technologies Cary 630 FT-IR. Liquid chromatography–mass spectrometry (LC-ESI-MS) was performed on an Agilent LC/Q-TOF instrument.

### Dynamic Light Scattering

Samples for DLS measurements were prepared by diluting 50 μL of aqueous NG samples in 1.5 mL DI water filtered using a 0.25 μm syringe filter. Samples were diluted/concentrated as needed, to give suitable concentrations for analysis. Measurements were taken in BrandTechMacro fluorescence disposable cuvettes at 25 °C, and analysis was conducted using the ZS Xplorer software. NG sizes are reported as the z-average ± standard deviation (nm), which is given through triplicate measurements. The polydispersity index is reported as PDI ± the standard deviation. To investigate the thermoresponsive properties of the NGs, 50 μL of the diluted aqueous solution was measured with low-volume quartz batch cuvette (3 mm × 3 mm) by Malvern Panalytical, UK. The NG was heated from 25 to 80 °C with increments of 5 °C and in a region of 10 °C determined by the VPTT with 1 °C increments. The sample was equilibrated for 120 s at each increment in temperature. For heating/cooling cycles measurements, the sample was heated to 80 °C and then cooled to 25 °C for 5 times. The size measurements were recorded after an equilibration time of 1 h.

### Electrophoretic Light Scattering

Samples for ELS measurements were prepared by diluting 50 μL of aqueous NG suspension in 1.5 mL DI water with pH 7.2. Measurements were taken in folded capillary zeta Cells by Malvern Panalytical, UK at 25 °C. ζ-potentials are reported as ζ-potential ± standard deviation (mV), and the average is given through triplicate measurements.

### Transmission Electron Microscopy

Five μL of the NG suspension in water was pipetted directly onto a continuous carbon film grid with 200 mesh copper (EM resolutions) and left on the grid for 1 min. Then the remainder of the solution was removed gently using a filter paper. This was repeated two more times. The grid was then stained with 5 μL of 2% (w/w) uranyl acetate stain for 1 min, after which the stain was removed gently using a filter paper. For images obtained at different temperatures, the samples were heated, then the samples were dropped on the carbon mesh directly. Cryo-TEM samples were prepared utilizing a plunge freezer (Leica EM GP2). Samples were prepared at 4 or 60 °C. In the plunge freezer, 3 μL of the sample were placed onto a lacey Formvar/Si monoxide carbon film grid with 300 mesh copper (TED PELLA, USA). The grid was then blotted on a filter paper, resulting in the formation of thin liquid films of 10–300 nm thickness freely spanning across the micropores. The sample grid assembly was rapidly vitrified with liquid ethane at −184 °C. The vitreous sample was kept under liquid nitrogen until it was loaded into a cryogenic sample holder (Gatan Single Tilt Cryo-Holder). All images were processed using ImageJ and magnifications are given in the captions.

### High Performance Liquid Chromatography

The column temperature was set at 40 °C. The mobile phase consisted of acetonitrile and triple distilled water (5/95 to 95/5, v/v), which was used for gradient elution at a flow rate of 0.5 mL/min. The injection volume was 20 μL. The eluent was monitored at 230 nm^70^ for detection of DTX over a period of 6 min. Analytical curves were constructed measuring the absorption of standard aqueous solutions of DTX (0, 0.5, 1, 5, 10, 50 μg/mL) at 230 nm. Calibration curve showed in Figure S23. The measurements were performed in triplicate for the quantification of DTX in the various experiments. Data acquisition and analysis were performed using Agilent OpenLab 2.8.

### Liquid Chromatography–Mass Spectrometry

Two μL of sample was injected onto the EclipsePlus C18 column 1.8 μm, 2.1 mm × 50 mm. A gradient of mobile phase A: 0.1% formic acid in water and B: 0.1% formic acid in acetonitrile was used. The mass spectrometer operated in positive and negative ionization mode with mass range 150 to 1500 *m*/*z*.

### General Synthetic Procedures

All reactions were carried out under a nitrogen atmosphere, with oven-dried glassware, and deoxygenated DI water which was filtrated and deoxygenated overnight prior to the synthesis.

### Synthesis of NGs

A solution of acrylamide (6.25 μL, 0.0350 mmol), PEG diacrylate (1.2 μL, 0.0040 mmol), and the monomer (TEGMA/DEGMA/MOEMA) (quantities given in Table S1) in 1 mL of DI water was bubbled with nitrogen and stirred for 30 min at room temperature. The reaction mixture was placed in an ice bath. Sodium dodecyl sulfate (SDS) (8.6 mg, 0.0300 mmol) was dissolved in 1 mL of DI water; the resulting solution was added to the reaction mixture and continuously stirred at 0 °C for 30 min. Ammonium persulfate (APS) (1.12 mg, 0.0005 mmol) and *N,N,N′,N′*-tetramethylethylenediamine (TEMED) (1.70 μL, 0.0110 mmol) were dissolved in 1 mL water and added to the reaction mixture. The solution was left to bubble for 1 h at 0 °C before quenching by exposure to air. The solution was washed and purified by centrifugation against DI water (2 mL × 3) at 1000 rpm for 10 min in a 30 kDa MWCO centrifugal unit, yielding purified aqueous solutions of the NG. The NG solution formed was removed, kept in a falcon tube/Eppendorf tube and stored in the fridge at 2–6 °C.

### Stability and Degradability Studies

For long-term storage studies, the NG sample (1.5 mg/mL) was stored at 2–6 °C for 100 d. DLS measurements were taken for size and ζ-potential at predetermined time points, and the sample was then returned to the fridge. For the degradability study, the NG sample (1.5 mg/mL) was placed in an Eppendorf tube, sealed with parafilm and incubated at 37 °C in a water bath. DLS measurements were taken for size and ζ-potential at predetermined time points, after which the sample was then returned to the water bath. For stability in media, 100 μL of the NG sample (1.5 mg/mL) were diluted with either water, DMEM, and DMEM supplemented with 10% FBS and left to equilibrate at room temperature for 15 min before taking a reading on the DLS (time 0 h). The samples were then incubated at 37 °C for 2 h and subsequently analyzed by DLS.

### Synthesis of Compound **1**

1-Ethyl-3-(3-(dimethylamino)­propyl)­carbodiimide (5 mg, 0.026 mmol) and mono-2-(methacryloyloxy)­ethyl succinate (6.4 μL, 0.030 mmol) were dissolved in 2.5 mL of dry DCM and cooled to 0 °C. DTX (20 mg, 0.025 mmol) and 4-(Dimethylamino)­pyridine (DMAP) (4 mg, 0.017 mmol) were dissolved in 1.5 mL of dry DCM. The DTX/DMAP solution was added dropwise to the reaction flask and stirred at 0 °C for 2 h. The reaction was stirred overnight at room temperature and then it was diluted with 1 mL of dry DCM, washed with 1 M HCl (3 × 3 mL) and with brine (1 × 3 mL). The organic phases were collected, dried over NaSO_4_, filtered and the solvent was evaporated in vacuum. The residue was dissolved in a minimum amount of DCM, subjected to preparative SiO_2_ TLC with dichloromethane and methanol (97:3 v/v) as the mobile phase. The dry silica containing the product was scraped off the plate, washed with ethyl acetate, filtered and the solvent was removed under reduced pressure and dried under vacuum to get compound **1** as a clear oil. (4.8 mg, 0.004 mmol, yield: 19%).

^1^H NMR (400 MHz, CDCl_3_), δ (ppm): 1.12 (*s*, 3H, H_ac_), 1.22 (*s*, 3H, H_ab_), 1.33 (*s*, 9H, H_*z*_), 1.65 (*s*, 2H, H_*y*_), 1.75 (*s*, 3H, H_*x*_), 1.94 (*s*, 6H, H_v_ and H_aa_), 2.42 (*s*, 3H, H_u_), 2.60 (*s*, 2H, H_t_), 2.71 (*m*, 4H, H_r_ and H_s_), 3.93 (*d*, 1H, H_q_), 4.19 (*s*, 2H, H_p_), 4.32 (*m*, 5H, H_n_ and H_o_), 4.96 (*d*, 1H, H_m_), 5.20 (*s*, 1H, H_l_), 5.34 (*s*, 1H, H_k_), 5.45 (*s*, 1H, H_j_), 5.60 (*s*, 1H, H_i_), 5.68 (*d*, 1H, H_h_), 6.12 (*s*, 1H, H_g_), 6.23 (*m*, 1H, H_f_), 7.31 (*m*, 3H, H_e_), 7.39 (*t*, 2H, H_d_), 7.50 (*t*, 2H, H_c_), 7.61 (*t*, 1H, H_b_), 8.11 (*d*, 2H, H_a_). TOF-MS-ES+ *m*/*z* calculated for C_53_H_65_O_19_Na_1_: 1042.40. Found [M + Na]+: 1042.40 and adducts. TLC: Rf = 0.33 (3% methanol in DCM).

### Synthesis of NGs with Compound **1**

A solution of acrylamide (6.25 μL, 0.035 mmol), PEG diacrylate (1.2 μL, 0.0040 mmol), the monomer (TEGMA) (0.0250 mmol) and **1** (0.6 mg, 0.0006 mmol) in 1 mL of DI water was bubbled with nitrogen and stirred for 30 min at room temperature. The reaction mixture was placed in an ice bath, and sodium dodecyl sulfate (SDS) (8.6 mg, 0.0300 mmol) was dissolved in 1 mL of DI water and the resulting solution was added to the reaction mixture and continuously stirred at 0 °C for 30 min. Ammonium persulfate (APS) (1.12 mg, 0.0005 mmol) and *N,N,N′,N′*-tetramethylethylenediamine (TEMED) (1.70 μL, 0.0110 mmol) were dissolved in 1 mL water and added to the reaction mixture. The solution was left to bubble for 1 h at 0 °C before quenching by exposure to air. The solutions were washed and purified by centrifugation against DI water (2 mL × 3) at 1000 rpm for 10 min in a 30 kDa MWCO centrifugal unit, yielding purified aqueous solutions of the NG. The NG solution formed was removed, kept in a falcon tube/Eppendorf tube and stored in the fridge at 2–6 °C.

### Encapsulation and Loading Efficiency Measurements

The encapsulation efficiency (EE%) and loading efficiency (LE%) of NG@TEGMA_DTX_covalent, NG@DTX_covalent and NG@TEGMA_DTX_mixed were measured using HPLC. The aqueous filtrates obtained from the NGs purification step were lyophilized overnight, then washed with 1:1 ethanol/DI water (v/v). The resulting solution was subjected to DTX quantitation via the measurement of the absorbance at 230 nm to quantify the nonencapsulated DTX. The EE% was further calculated using the following formula, eq 1:

1 $$\mathrm{EE}\%=\frac{\left(\mathrm{DTX total}\right)-\left(\mathrm{DTX HPLC}\right)}{\left(\mathrm{DTX total}\right)}\times 100$$

The nanoparticle suspensions were lyophilized over 48 h and the resulting powders accurately weighed. To calculate the LE%, the following formula was used, eq 2:

2 $$\mathrm{LE}\%=\frac{\left(\mathrm{mass DTX loaded}\right)}{\left(\mathrm{mass empty NGs}\right)}\times 100$$

### Drug Release Studies at 37 °C

Aqueous suspensions of DTX-loaded NGs (2 mL) were placed into Eppendorf tubes, sealed with parafilm, and incubated at 37 °C. At fixed time point, 200 μL of the samples were removed, centrifuged in centrifugal filters (MWCO 100 kDa, 5000 rcf, 3 min, 37 °C) and the resulting filtrates were collected. The released DTX was quantified from the individual filtrates allowing for the construction of cumulative release curves. The data is reported as the% ± standard deviation, which is given through triplicate measurements, where the averages are reported.

### Drug Release Studies with Increasing Temperature

Aqueous suspensions of DTX-loaded NGs (2 mL) were placed into glass vials and heated from 25 to 65 °C with 5 °C intervals and equilibration of 2 min. The temperature was monitored with a probe. At each interval, 100 μL of the samples were removed and substituted with 100 μL of DI water, centrifuged in centrifugal filters (MWCO 100 kDa, 5000 rcf, 3 min, 25 °C) and the resulting filtrates were collected. The released DTX was quantified from the individual filtrates allowing for the construction of cumulative release curves. The data is reported as the% ± standard deviation, which is given through triplicate measurements, where the averages are reported.

### Cell Culture Preparation

HeLa cells were cultured in Dulbecco’s modified Eagle’s medium (DMEM) and supplemented with 10% fetal bovine serum (FBS). All cells were maintained at 37 °C under 5% CO_2_ atmosphere.

### Cell Viability *In Vitro*

HeLa cells were seeded in 96-well plates (5000 cells/well) in DMEM supplemented with 10% FBS (100 μL/well). Blank wells contained culture medium only and control wells had cells with no NG. Following incubation for 48 h at 37 °C with 5% CO_2_, the cell media was replaced (10 μL), and various concentrations of aqueous NG solutions (10 μL) were added to the cells. The cells were incubated for 24 h at 37 °C with 5% CO_2_. Ten μL in each well was removed and replaced with cell viability stain solution (CCK-8) (10 μL) and incubated for 2 h at 37 °C with 5% CO_2_. The optical density of the produced stain was monitored at 450 nm using a SpectraMax ABS microplate reader. Untreated cells (without any NGs added) were used as a negative control. Cell viability was determined using the following eq 3:

3 $$\mathrm{Cell viability (\%)}=\frac{\left(\mathrm{absorbance of test well}\right)-\left(\mathrm{absorbance of blank well}\right)}{\left(\mathrm{absorbance of control well}\right)-\left(\mathrm{absorbance of blank well}\right)}\times 100$$

where absorbance is the optical absorbance at 450 nm.

### Cellular Uptake

HeLa cells were seeded in μ-Slide 8 well chambered slides (Ibidi, GmbH, Germany) plate (25000 cells/well) and incubated for 48 h. After 48 h, 5 μL of media were replaced with 5 μL of a 150 μg/mL solution of fluorescein-labeled NG at the following incubation time points: 24 h, 8 and 2 h before fixing. The final NG concentration in the cells was around 3 μg/mL. The positive control was added at 24 h before staining. The media was removed and the cells were washed thrice with phosphate-buffered saline (PBS), fixed with paraformaldehyde (PFA) solution for 15 min, washed with PBS three times, stained with Hoechst solution for 15 min (1 μL/mL in PBS), washed thrice with PBS and stored in PBS. The cells were then imaged using the Leica Stellaris 8 STED-FALCON inverted microscope (Leica, Germany) using LASX software (Leica, Germany). Images were analyzed using ImageJ (NIH, USA) and quantification was carried out following the protocol by Shihan et al.^69^

### Statistical Analysis

All statistical analyses were performed using SciPy. The *p* values were obtained by student’s unpaired *t* test, and the *p* value of <0.05 was considered statistically significant. The data is presented as mean ± standard deviation with *n* = 3 of biological replicates.

## Supplementary Material



## Data Availability

The data supporting the findings of this study are available within the article and Supporting Information.

## Notes

FT-IR spectra of the starting materials and NGs. ^1^H NMR spectra of **1**, DTX, and the mono-2-(methacryloyloxy)­ethyl succinate. Tables with molar quantities of the formulations, DLS, and ELS data. Flory–Rehner equation and derived structural network parameters. Negative stained (NS) TEM of size changes with temperature. NS-TEM for degradability experiment. LC-ESI-MS spectra. Drug calibration curves from HPLC. (PDF)
