# Supplementary material for: Precision Temperature Control of Volume Phase Transition in Biocompatible PEG-Based Nanogels for Triggered Drug Release
Source: ACS Nano Med. 2026 Jun 10;1(6):1469–83. doi: 10.1021/acsnanomed.6c00111 (PMC13352944; doi:10.1021/acsnanomed.6c00111)
Supplement: Supplementary file 1 [file nm6c00111_si_001.pdf]

SUPPORTING INFORMATION

Precision Temperature Control of Volume Phase Transition in Biocompatible PEG Based Nanogels for Triggered Drug Release

Sofia Patri[a,d]*, Saanchi Agrawala*[b]*, Paul Joseph Kempen*[c]*, Nguyen Thi Kim Thanh**[d,e]*, Nazila Kamaly**[b]*.*

[a] Department of Materials Imperial College London, London, SW7 2AZ, United Kingdom.

[b] Department of Chemistry Imperial College London, London, SW7 2AZ, United Kingdom, nazila.kamaly@imperial.ac.uk.

[c] National Center for Nano Fabrication and Characterization, Technical University of Denmark, Kgs Lyngby, 2800, Denmark.

[d] Healthcare Biomagnetics and Nanomaterials Laboratories, UCL, London, W1S 4BS, United Kingdom, ntk.thanh@ucl.ac.uk.

[e] Biophysics Group, Department of Physics and Astronomy, University College London, London WC1E 6BT, United Kingdom, ntk.thanh@ucl.ac.uk.

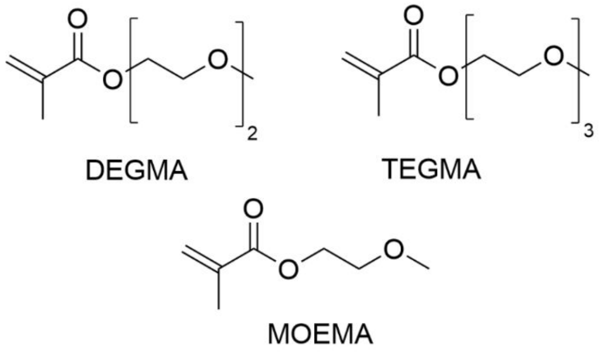

Figure S. 1: Chemical structure of selected thermo-responsive monomers: di(ethylene glycol) methyl ether methacrylate (DEGMA), tri(ethylene glycol) methyl ether methacrylate (TEGMA) and 2-methoxyethyl methacrylate (MOEMA).

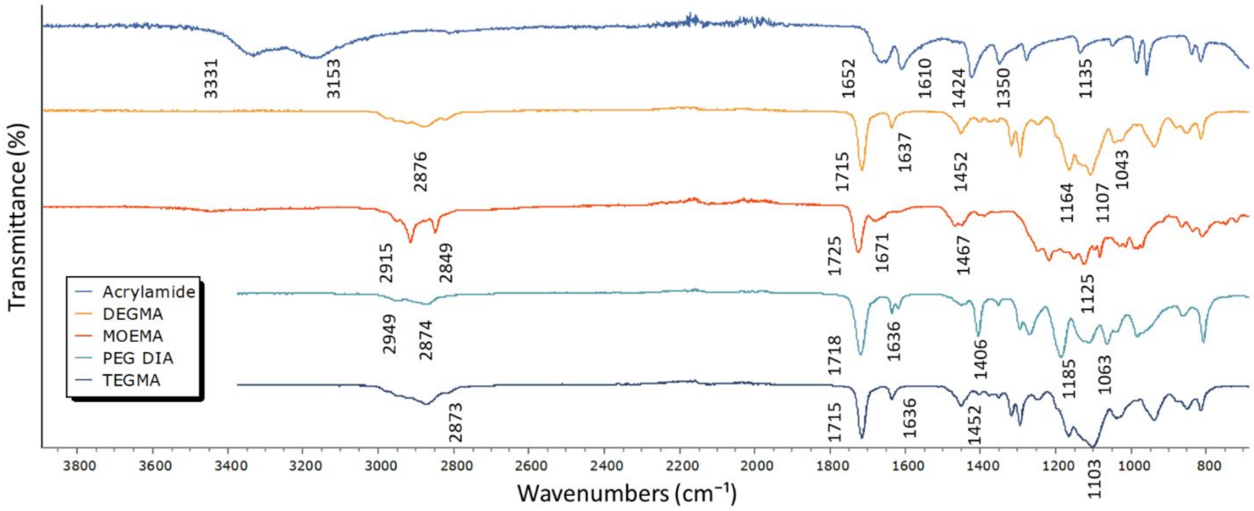

Figure S. 2: Fourier transform infrared spectroscopy (FT-IR) spectra of the NGs starting materials: acrylamide, di(ethylene glycol) methyl ether methacrylate (DEGMA), 2-methoxyethyl methacrylate (MOEMA), poly(ethylene glycol) diacrylate with average Mn 250 (PEG DIA), tri(ethylene glycol) methyl ether methacrylate (TEGMA). Signals (cm^{-1}) are assigned as follows: 3331 (broad) NH stretching (amine), 2876/2874/2873/2849 (sharp) CH_2 stretching (alkyl sp^3), 1725/1718/1715 (sharp) C=O stretching (carbonyl), 1671/1652 (sharp) C=O stretching (carbonyl), 1637/1636/1610 (sharp) C=C stretching, 1467/1452/1424/1406 (sharp) CH bending, 1185 to 1063 (broad) C-O-C stretching.

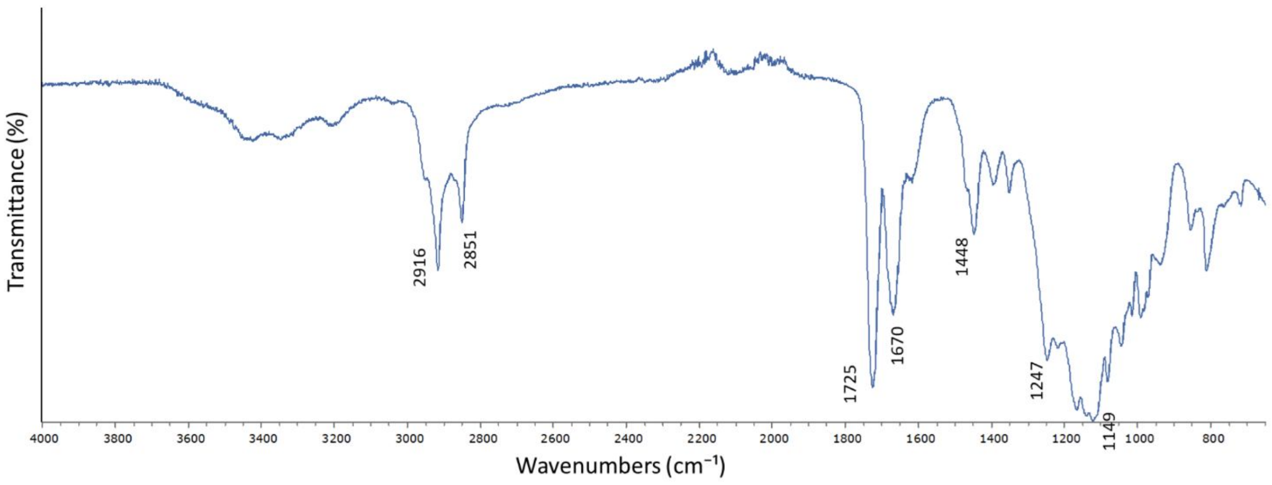

Figure S. 3: Fourier transform infrared spectroscopy (FT-IR) spectrum of NG@Control. Signals (cm^{-1}) are assigned as follows: 2916 and 2851 (sharp) CH_2 stretching (alkyl sp^3), 1725 and 1670 (sharp) C=O stretching (carbonyl), 1448 (sharp) CH bending, 1149 (broad) C-O-C stretching.

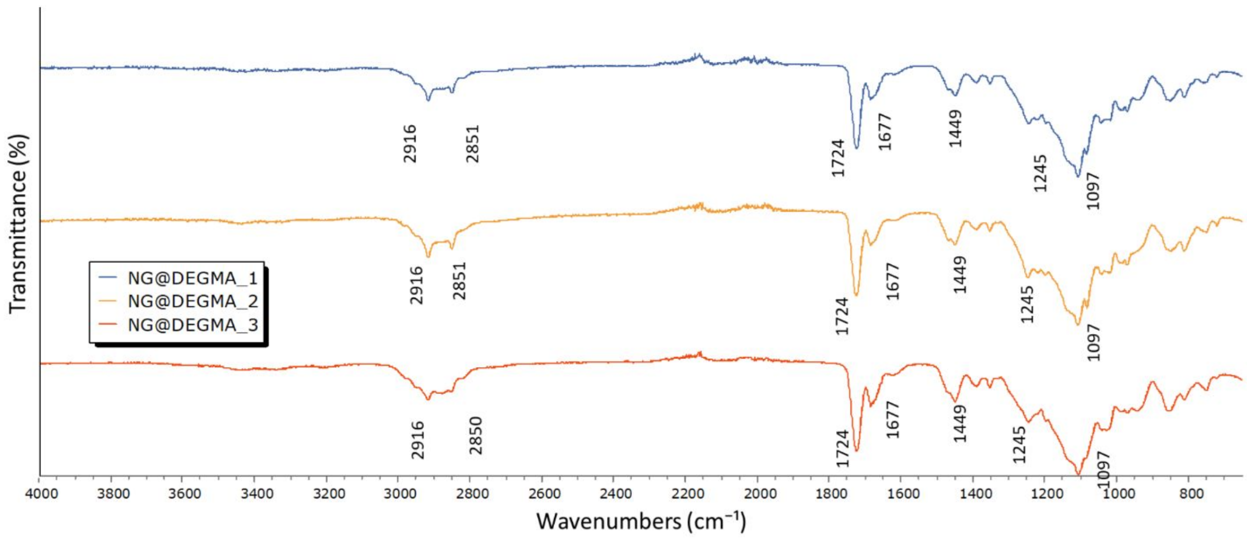

Figure S. 4: Fourier transform infrared spectroscopy (FT-IR) spectra of NG@DEGMA 1 to 3. Signals (cm^{-1}) are assigned as follows: 2916 and 2851 (sharp) CH_2 stretching (alkyl sp^3), 1724 and 1677 (sharp) C=O stretching (carbonyl), 1449 (sharp) CH bending, 1097 (broad) C-O-C stretching.

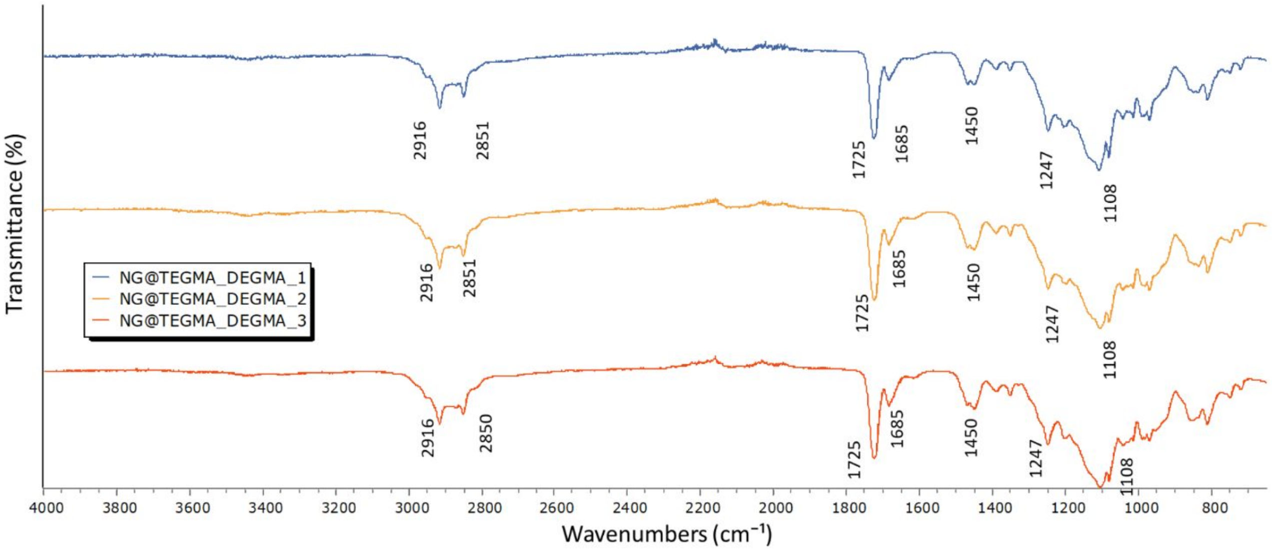

Figure S. 5: Fourier transform infrared spectroscopy (FT-IR) spectra of NG@TEGMA_DEGMA 1 to 3. Signals (cm^{-1}) are assigned as follows: 2916 and 2851 (sharp) CH_2 stretching (alkyl sp^3), 1725 and 1685 (sharp) C=O stretching (carbonyl), 1450 (sharp) CH bending, 1108 (broad) C-O-C stretching.

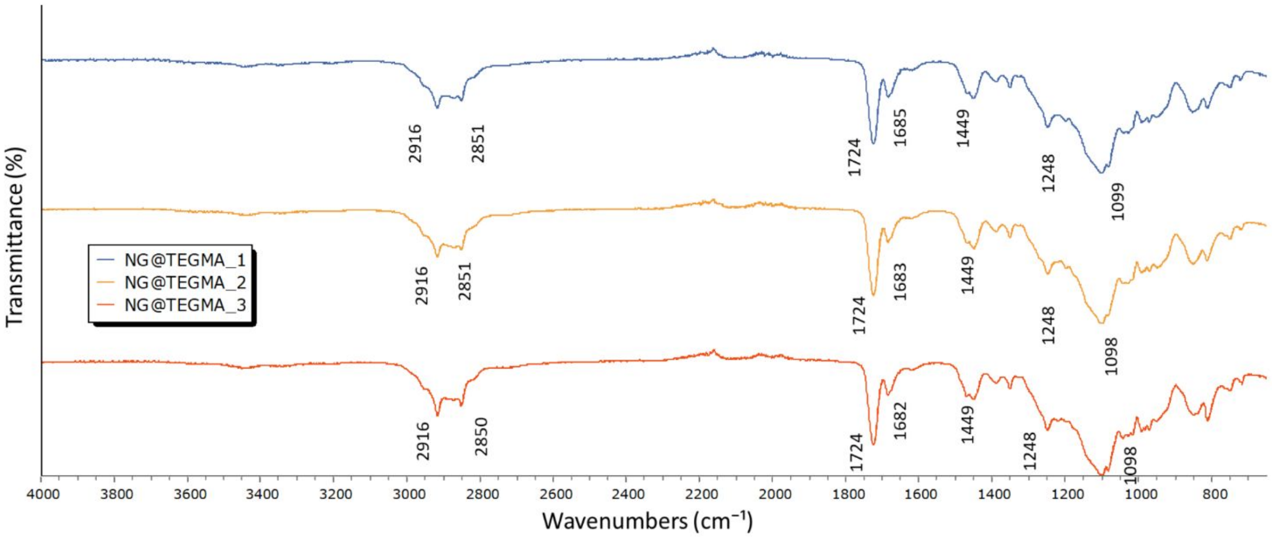

Figure S. 6: Fourier transform infrared spectroscopy (FT-IR) spectra of NG@TEGMA 1 to 3. Signals (cm^{-1}) are assigned as follows: 2916 and 2851 (sharp) CH_2 stretching (alkyl sp^3), 1724 and 1685/1683/1682 (sharp) C=O stretching (carbonyl), 1449 (sharp) CH bending, 1099/1098 (broad) C-O-C stretching.

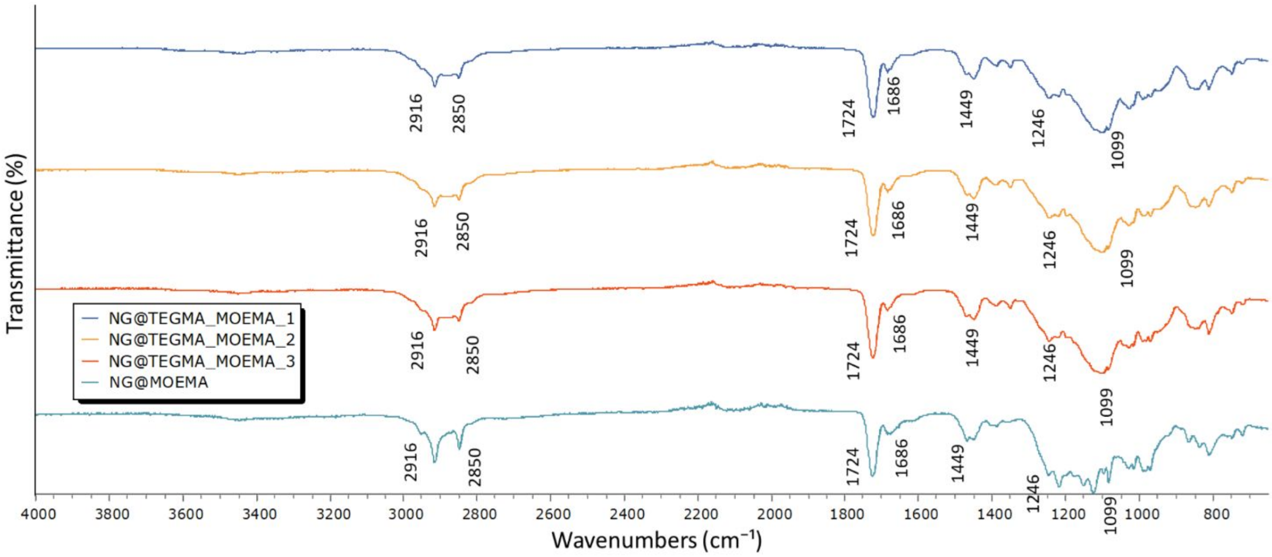

Figure S. 7: Fourier transform infrared spectroscopy (FT-IR) spectra of NG@TEGMA_MOEMA 1 to 3 and NG@MOEMA. Signals (cm^{-1}) are assigned as follows: 2916 and 2850 (sharp) CH_2 stretching (alkyl sp^3), 1724 and 1686 (sharp) C=O stretching (carbonyl), 1449 (sharp) CH bending, 1099 (broad) C-O-C stretching.

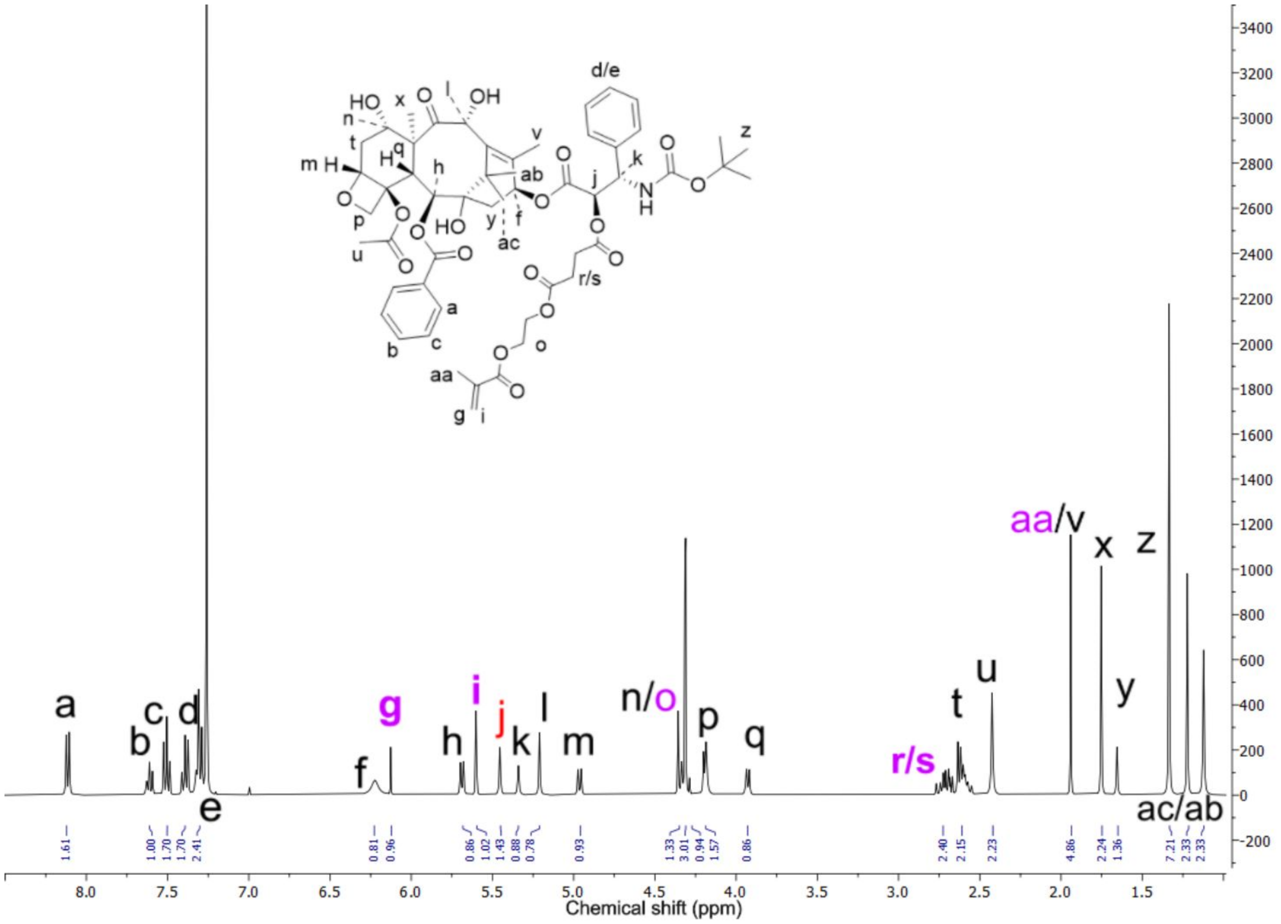

Figure S. 8: ^{1}H-NMR spectrum of **1** ($CDCl_3$, 400 MHz). Peaks labeled in magenta correspond to the succinate moiety (cf. **Figure S. 9**), while those in black are attributed to the docetaxel (cf. **Figure S. 10**). The resonance for proton *j* is highlighted in red to indicate the significant downfield shift resulting from the covalent conjugation of the two molecules.

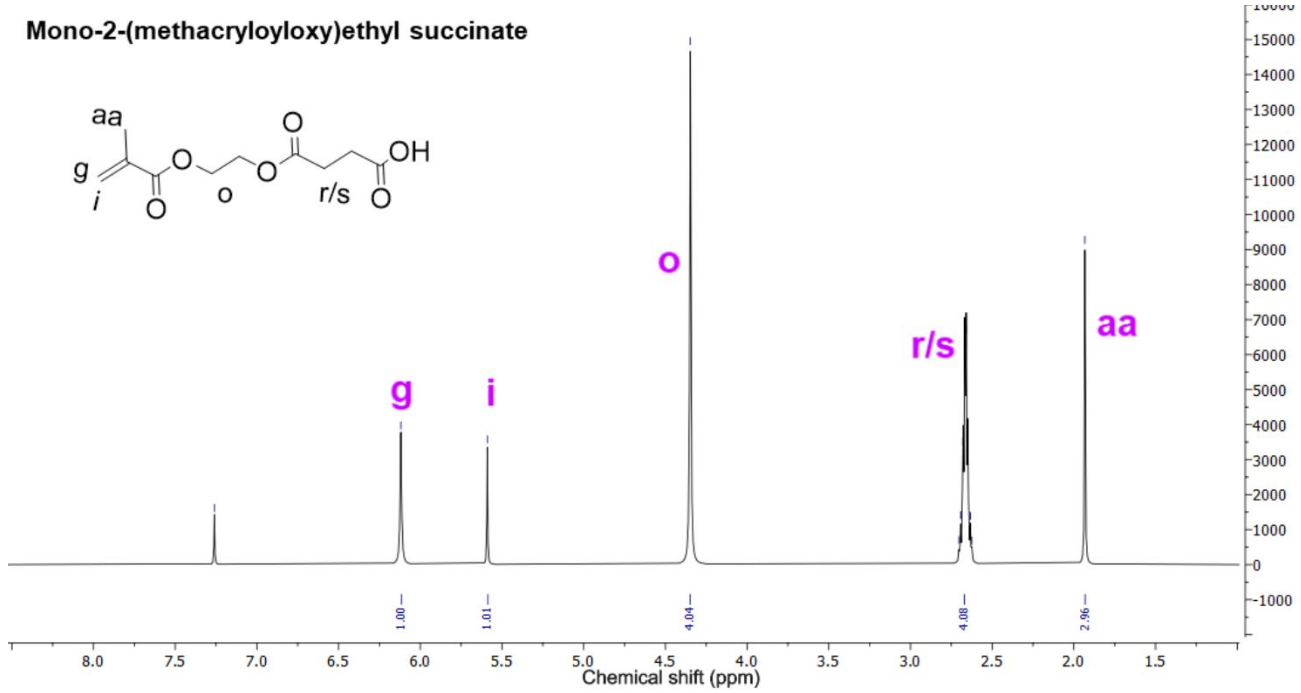

Figure S. 9: ^{1}H-NMR spectrum of mono-2-(methacryloyloxy)ethyl succinate (peaks assignments as from ref[1]) ($CDCl_3$, 400 MHz).

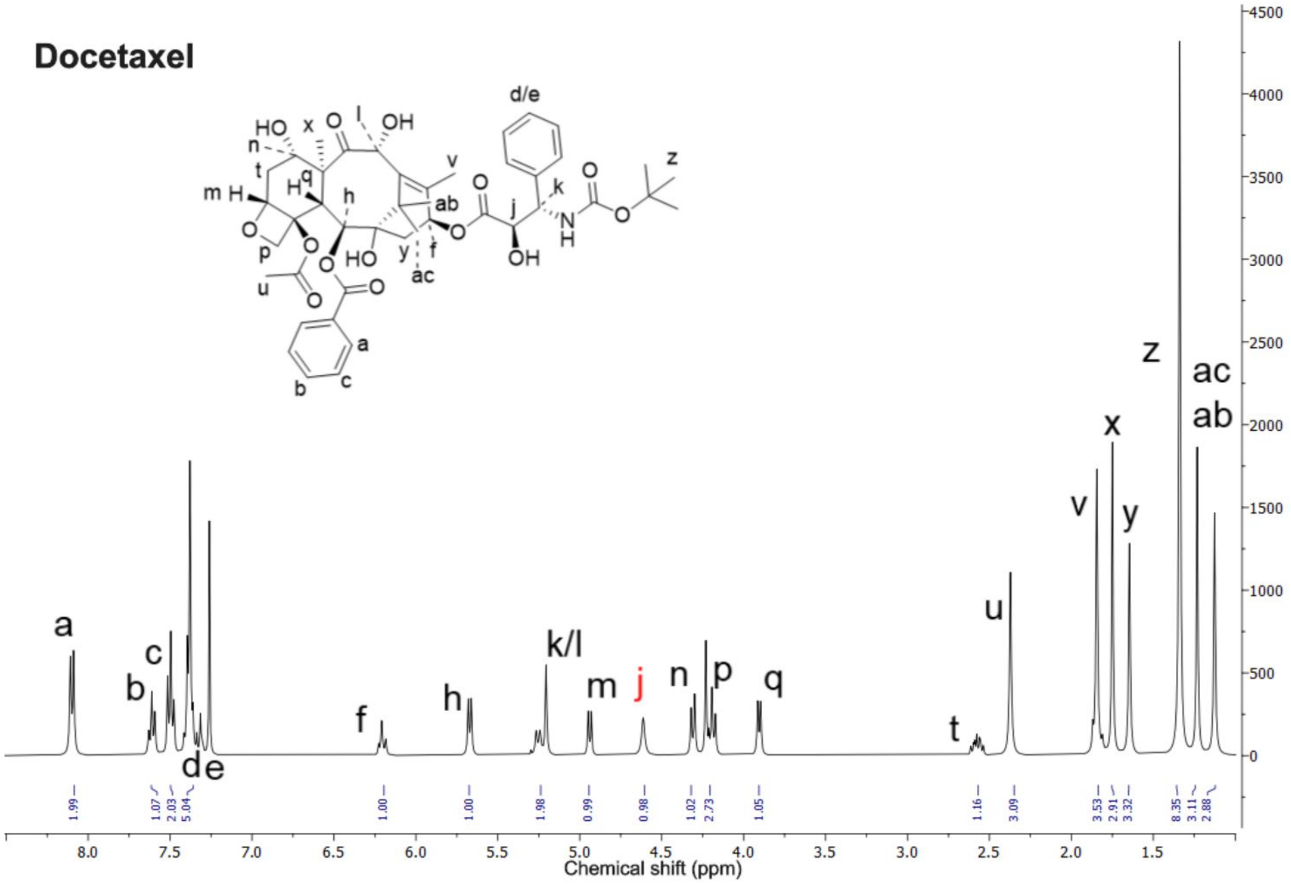

Figure S. 10: ^{1}H-NMR spectrum of docetaxel (peaks assignments as from ref[2]) ($CDCl_3$, 400 MHz).

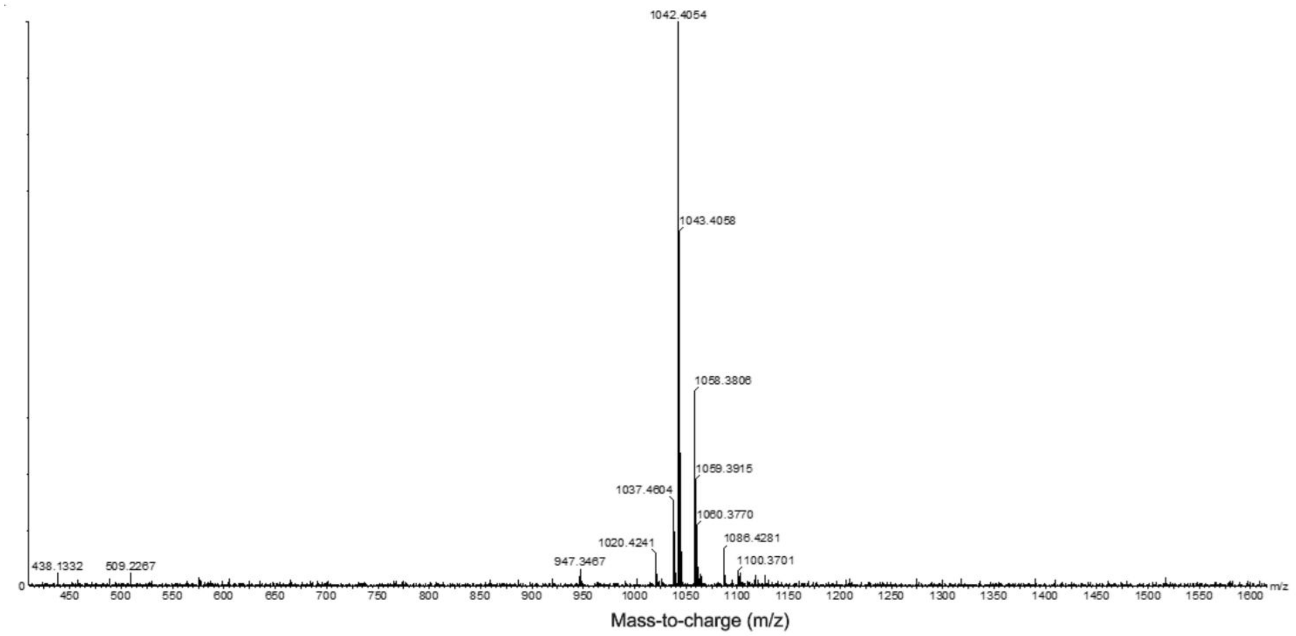

Figure S. 11: Mass spectrum of **1** showing the elemental peak at 1042 corresponding to [M + Na]+.

Table S. 1: Formulations of NGs and ζ-potential.

Sample	TEGMA (mmol)	DEGMA (mmol)	MOEMA (mmol)	ζ-potential (mV)
NG@Control	-	-	-	-0.6 ± 0.1
NG@DEGMA_1	-	0.015	-	-36.0 ± 2.0
NG@DEGMA_2	-	0.020	-	-31.0 ± 1.0
NG@DEGMA_3	-	0.025	-	-28.0 ± 1.0
NG@TEGMA_1	0.015	-	-	-29.0 ± 6.0
NG@TEGMA_2	0.020	-	-	- 30.0 ± 11.0
NG@TEGMA_3	0.025	-	-	-25.0 ± 4.0
NG@TEGMA_ DEGMA_1	0.008	0.017	-	-22.0 ± 1.0
NG@TEGMA_ DEGMA_2	0.0125	0.0125	-	-20.0 ± 2.0
NG@TEGMA_ DEGMA_3	0.017	0.008	-	-23.0 ± 7.0
NG@TEGMA_ MOEMA_1 (60 : 40)	0.015	-	0.01	-18.0 ± 2.0
NG@TEGMA_ MOEMA_2 (70 : 30)	0.017	-	0.008	-17.0 ± 7.0
NG@TEGMA_ MOEMA_3 (80 : 20)	0.019	-	0.005	-27.0 ± 1.0
NG@MOEMA	-	-	0.025	-20.0 ± 6.0

Note: All NGs synthesis were carried out with AM (0.035 mmol) and PEG diacrylate (0.004 mmol) as co-monomers. The samples are named after the thermo-responsive monomers used. The subscripts in the sample's name are used to distinguish formulations with the same monomers, but different molar quantities, as detailed in the table.

Table S. 2: Characterization of NGs before and after the VPTT.

Sample	D hydro (nm) at 25 °C	PDI at 25 °C	VPTT (°C)	D hydro (nm) after VPTT	PDI after VPTT	ζ-potential (mV) after VPTT
NG@DEGMA_1	249 ± 51	0.8 ± 0.1	43	59 ± 6	0.08	-20 ± 4
NG@DEGMA_2	241 ± 61	0.9 ± 0.1	40	44 ± 2	0.01	-24 ± 8
NG@DEGMA_3	303 ± 15	0.8 ± 0.2	34	55 ± 1	0.01	-14 ± 1
NG@TEGMA_1	266 ± 54	0.6 ± 0.2	59	88 ± 6	0.06	-36 ± 4
NG@TEGMA_2	317 ± 41	0.4 ± 0.1	55	83 ± 6	0.10	-24 ± 2
NG@TEGMA_3	272 ± 45	0.4 ± 0.1	52	98 ± 4	0.09	-34 ± 4
NG@TEGMA_DEGMA_1	219 ± 53	0.4 ± 0.2	64	41 ± 1	0.17	-15 ± 1
NG@TEGMA_DEGMA_2	253 ± 39	0.4 ± 0.1	65	47 ± 1	0.07	-23 ± 3
NG@TEGMA_DEGMA_3	309 ± 40	0.6 ± 0.1	66	49 ± 1	0.14	-27 ± 1
NG@TEGMA_ MOEMA_1 (60 : 40)	310 ± 70	0.5 ± 0.1	37	87 ± 1	0.09	-24 ± 2
NG@TEGMA_ MOEMA_2 (70 : 30)	266 ± 54	0.27 ± 0.1	47	32 ± 1	0.10	-26 ± 1
NG@TEGMA_ MOEMA_3 (80 : 20)	276 ± 13	0.2 ± 0.1	52	31 ± 1	0.09	-20 ± 4
NG@MOEMA	37 ± 2	0.2 ± 0.0	-	-	-	-25 ± 2

Note: The VPTT depends on the formulation as detailed in Table S. 1.

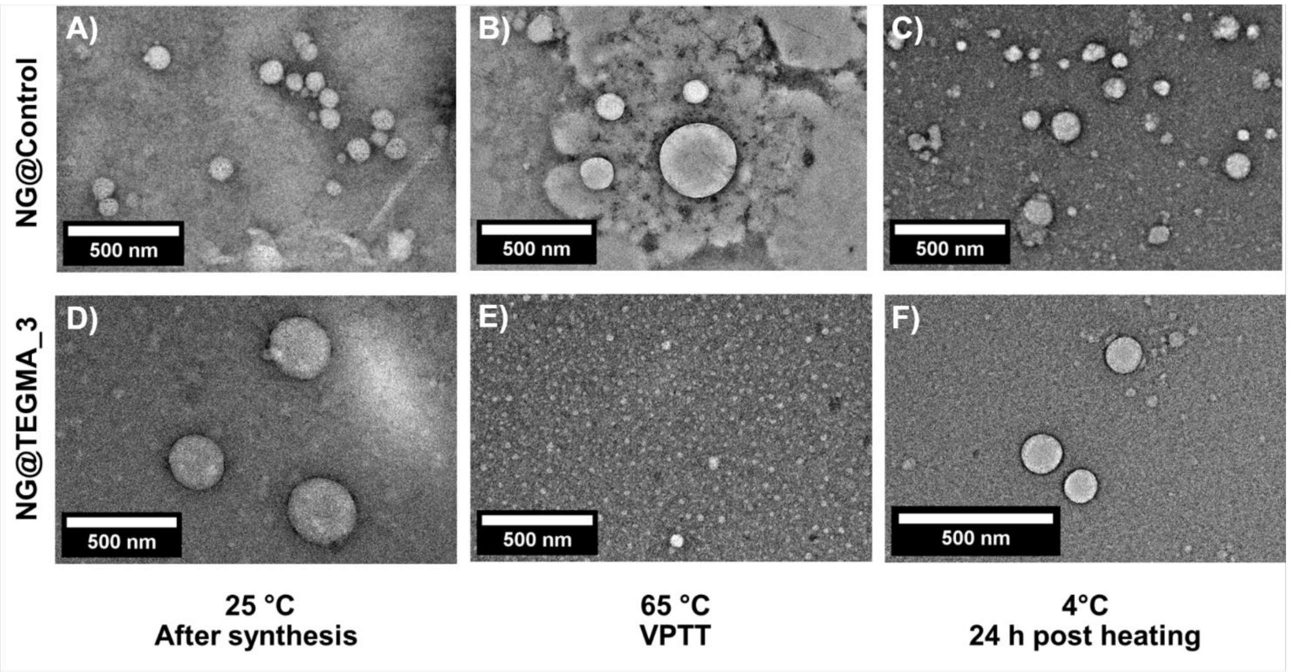

Figure S. 12: Transmission electron microscopy (TEM) images of: A) NG@Control at 25 °C, 15 kx, scale bar 500 nm; B) NG@Control at 65 °C, 15 kx, scale bar 500 nm; C) NG@Control at 4 °C, 24 h post heating, 15 kx, scale bar 500 nm; D) NG@TEGMA_3 at 25 °C, 20 kx, scale bar 500 nm; E) NG@TEGMA_3 at 65 °C (above VPTT), 20 kx, scale bar 500 nm; F) NG@TEGMA_3 at 4 °C, 24 h post heating, 20 kx, scale bar 500 nm. All samples were negatively stained (NS) with uranyl acetate and recorded with accelerating voltage (AV): 200 kV.

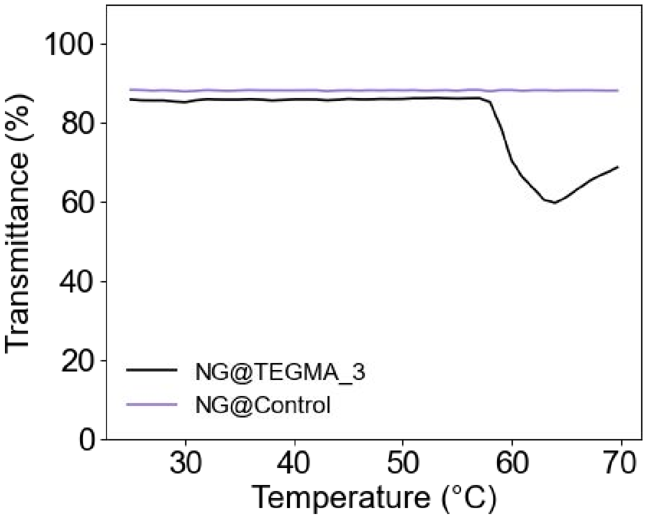

Figure S. 13: Temperature-dependent turbidity (transmittance) profile of NG@TEGMA_3. VPTT is around 58 °C, with a decrease in transmittance from ~86% to 60%, followed by a recovery at higher temperatures (T > 64 °C). The recovery is consistent with re-dispersion of the collapsed NGs. A non-responsive control is shown in purple for comparison.

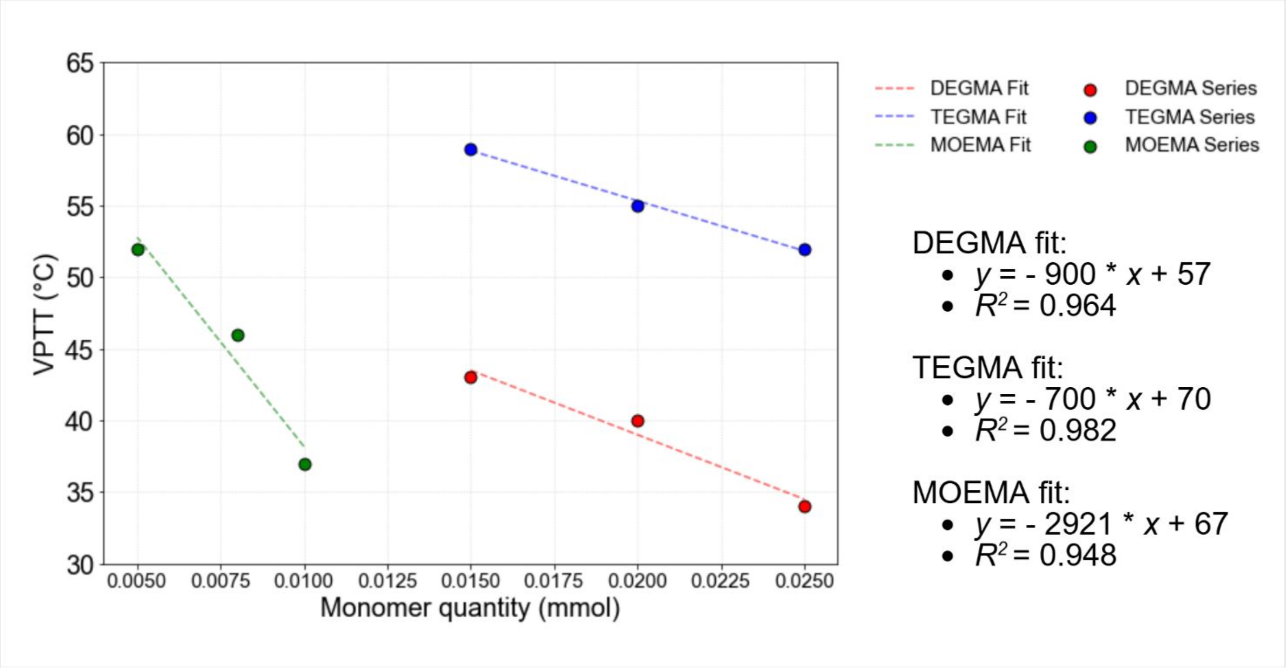

Figure S. 14. Modulation of the volume phase transition temperature VPTT via monomer quantity stoichiometry. Linear regression analysis of the VPTT as a function of the molar quantity (mmol) of OEGMA building blocks in the initial synthesis.

Equation S. 1. The average molecular weight between crosslinks (M_c) of the cross-linked matrix calculated using the Flory−Rehner equation[3,4]:

$$\overline{M_c} = -\frac{\rho_p V_1\left({\varphi_2}^{1/3} - \frac{\varphi_2}{2}\right)}{\ln(1-\varphi_2) + \varphi_2 + \chi\varphi_2^2} \qquad \textbf{(S.1)}$$

Where M_c is the average molecular weight between the cross-link, ρ_p is the density of the polymer (1.1 g/cm^3 for OEGMA-based systems[4]), V_1 is the molar volume of the solvent (water, 18.02 cm^3/mol), φ_2 is the volume fraction of the polymer in the gel swollen to equilibrium calculated as $\left(\frac{(D_{hydro}\ 25\ °\mathrm{C})}{(D_{hydro}\ \text{after VPTT})}\right)^3$ (**Equation S. 2**), χ is the polymer−solvent interaction parameter (PEG−water system, 0.45)[4].

Table S. 3. Structural network parameters of NGs derived from Flory–Rehner Analysis.

Sample	D hydro (nm) at 25 °C	D hydro (nm) after VPTT	Swelling Ratio (SD)	Vol. Fraction (ϕ2)	Mc (x 106 g/mol)
NG@DEGMA_1	249	59	4.22	0.013	0.61
NG@DEGMA_2	241	44	5.48	0.006	2.09
NG@DEGMA_3	303	55	5.51	0.006	2.13
NG@TEGMA_1	266	88	3.02	0.036	0.08
NG@TEGMA_2	317	83	3.82	0.018	0.30
NG@TEGMA_3	272	98	2.78	0.047	0.05
NG@TEGMA_DEGMA_1	219	41	5.34	0.007	1.86
NG@TEGMA_DEGMA_2	253	47	5.38	0.006	1.94
NG@TEGMA_DEGMA_3	309	49	6.31	0.004	4.27
NG@TEGMA_ MOEMA_1 (60 : 40)	310	87	3.56	0.022	0.20
NG@TEGMA_MOEMA_2 (70 : 30)	266	32	8.31	0.002	17.20
NG@TEGMA_MOEMA_3 (80 : 20)	276	31	8.9	0.001	24.84

Note: The linear swelling ratio (S_D) was calculated as ($\frac{(D_{hydro}\ 25\ °\text{C})}{(D_{hydro}\ \text{after VPTT})}$), **Equation S. 3**.[5] The polymer volume fraction (ϕ_2) and the number average molecular weight between crosslinks (M_c) were estimated using the Flory–Rehner equation (**Equation S. 1**) for networks prepared in the presence of a solvent.

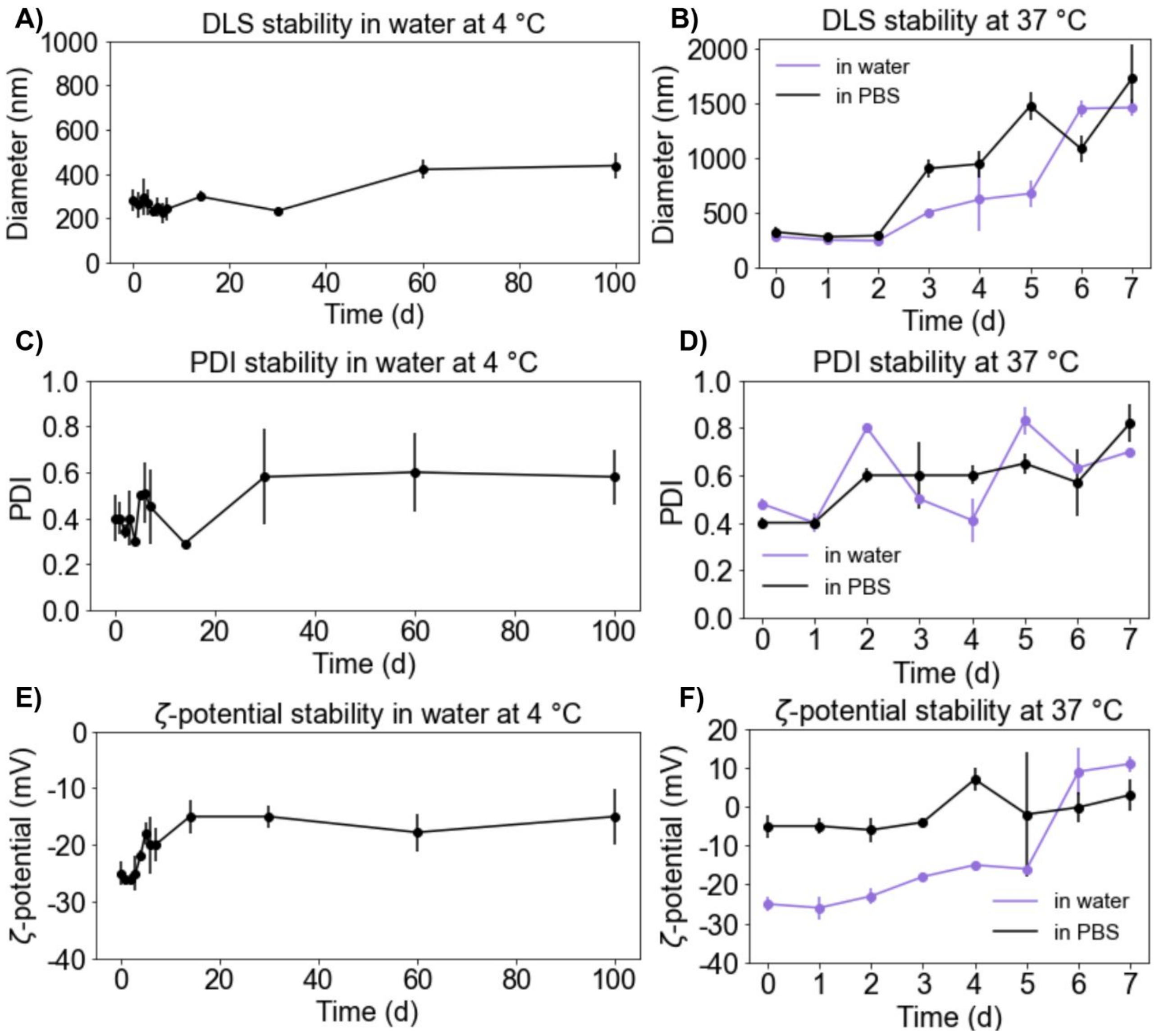

Figure S. 15: Characterization of NGs: A) hydrodynamic diameter of NG@TEGMA_3 stored at 4 °C for 100 d; B) hydrodynamic diameter of NG@TEGMA_3 incubated at 37 °C in DI water and phosphate-buffered saline (PBS) for 7 d; C) polydispersity (PDI) of NG@TEGMA_3 stored at 4 °C for 100 d; D) PDI of NG@TEGMA_3 incubated at 37 °C in DI water and PBS for 7 d; E) ζ-potential measurements of NG@TEGMA_3 stored at 4 °C for 100 d; F) ζ-potential measurements of NG@TEGMA_3 incubated at 37 °C in DI water and PBS for 7 d. In all cases, data are presented as mean ± standard deviation for n = 3.

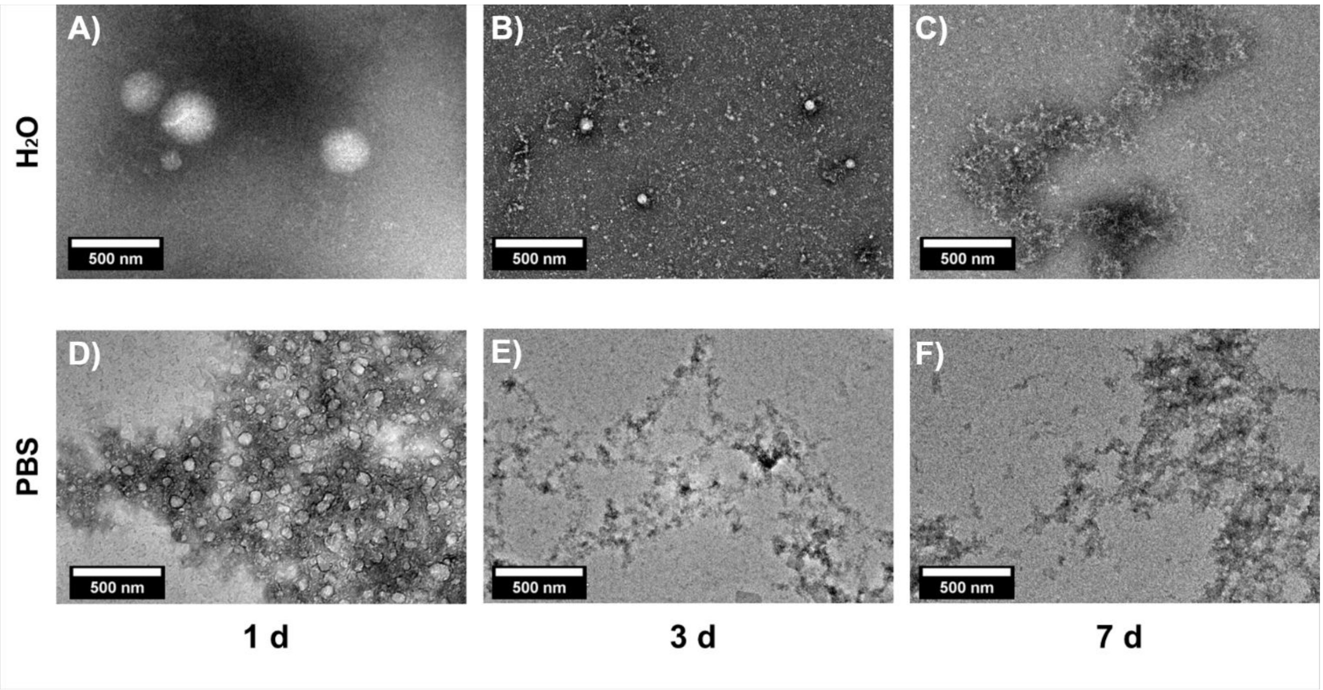

Figure S. 16: Transmission electron microscopy (TEM) images of: A) NG@TEGMA_3 at 37 °C in H_2O, 1 d, 12 kx, scale bar 500 nm; B) NG@TEGMA_3 at 37 °C in H_2O, 3 d, 12 kx, scale bar 500 nm; C) NG@TEGMA_3 at 37 °C in H_2O, 7 d, 12 kx, scale bar 500 nm; D) NG@TEGMA_3 at 37 °C in phosphate-buffered saline (PBS), 1 d, 12 kx, scale bar 500 nm; E) NG@TEGMA_3 at 37 °C in PBS, 3 d, 12 kx, scale bar 500 nm; F) NG@TEGMA_3 at 37 °C in PBS, 7d , 12 kx, scale bar 500 nm. All samples were negatively stained (NS) with uranyl acetate and recorded with accelerating voltage (AV): 200 kV.

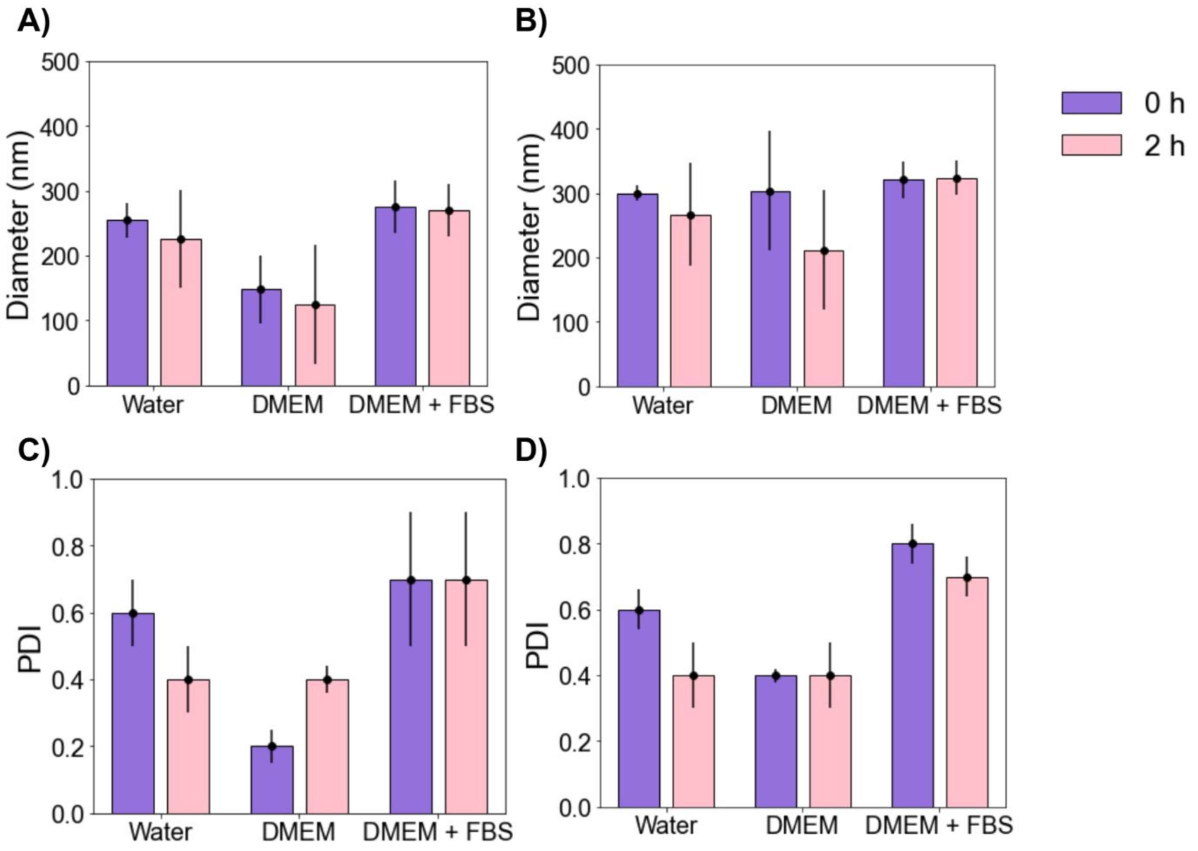

Figure S. 17. Characterization of NGs at time 0 h and upon 2 h incubation at 37 °C in DI water, DMEM, and DMEM supplemented with 10% FBS: A) hydrodynamic diameter of NG@Control; B) hydrodynamic diameter of NG@TEGMA_3; C) polydispersity (PDI) measurements NG@Control; D) PDI measurements NG@TEGMA_3. All the results are presented in triplicate, along with error bars, as mean ± SD (n = 3).

Table S. 4: Hydrodynamic diameter and polydispersity (PDI) before and after volume phase transition temperature (VPTT) in DMEM supplemented with 10% FBS.

Sample	D hydro (nm) before VPTT	PDI before VPTT	VPTT (°C)	D hydro (nm) after VPTT	PDI after VPTT
NG@TEGMA_1	361 ± 33	0.5	45	1359 ± 89	0.6
NG@TEGMA_2	399 ± 22	0.6	42	1110 ± 82	0.6
NG@TEGMA_3	391 ± 45	0.7	40	1156 ± 86	0.6

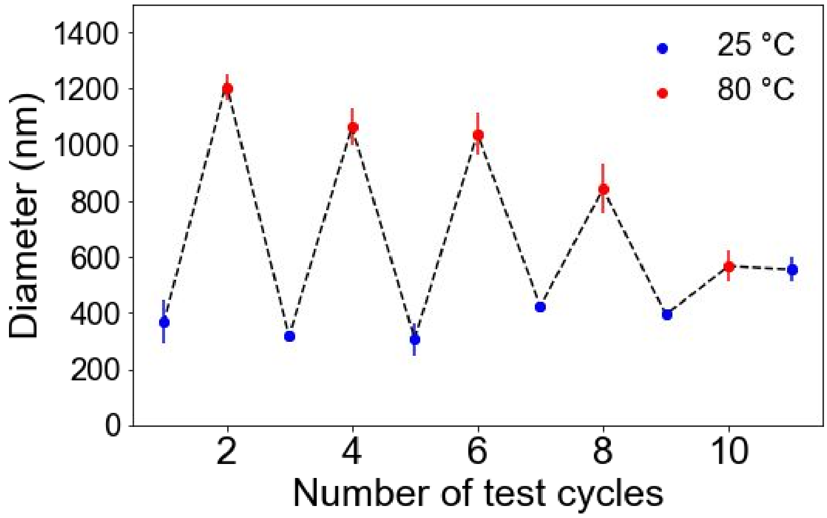

Figure S. 18: Cyclic measurement in DMEM supplemented with 10% FBS for NG@TEGMA_3. Equilibration time is 1 h per data point. In all cases, data are presented as mean ± standard deviation for n= 3 of biological replicates.

Table S. 5: Characterization of NGs for docetaxel (DTX) delivery with encapsulation (EE%) and loading efficiency (LE%) of docetaxel within the NGs.

Sample	D_{hydro} (nm)	PDI	ζ-potential (mV)	EE%	LE%
NG@TEGMA_ DTX_covalent	291 ± 28	0.35 ± 0.03	-11 ± 1	93 ± 4	17 ± 5
NG@TEGMA_DTX_ mixed	300 ± 3	0.39 ± 0.14	-23 ± 2	53 ± 18	7 ± 1
NG@DTX_covalent	162 ± 2	0.29 ± 0.03	5 ± 0.8	93 ± 4	55 ± 2

Table S. 6: Characterization of NGs for docetaxel (DTX) delivery after volume phase transition temperature (VPTT) at 52 °C.

Sample	D_{hydro} (nm) after VPTT	PDI after VPTT	ζ-potential (mV) after VPTT
NG@TEGMA_DTX_covalent	131 ± 6	0.05	-15 ± 1

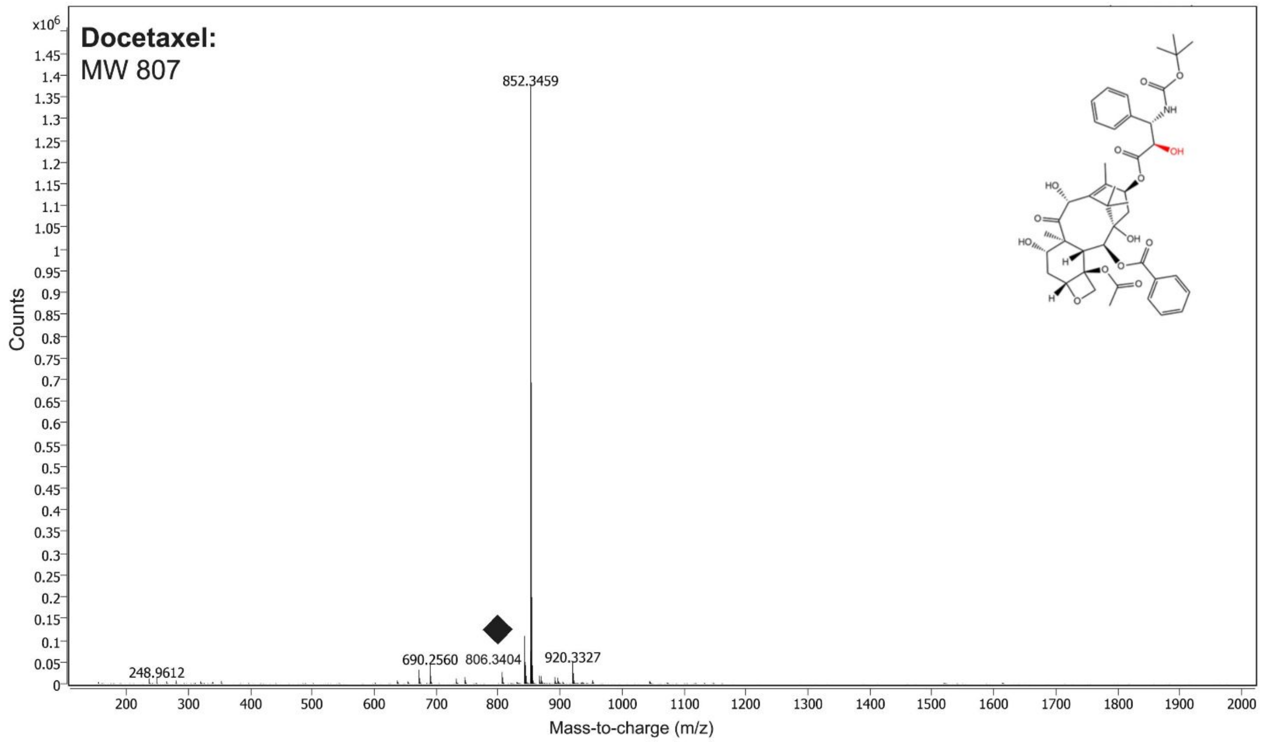

Figure S. 19: LC-ESI-MS spectrum and chemical structure of DTX; molecular ion theoretical mass for $[M_1 - H]^-$: 806.3393. The molecular ion is indicated with black diamond. $M_1 = C_{43}H_{53}NO_{14}$.

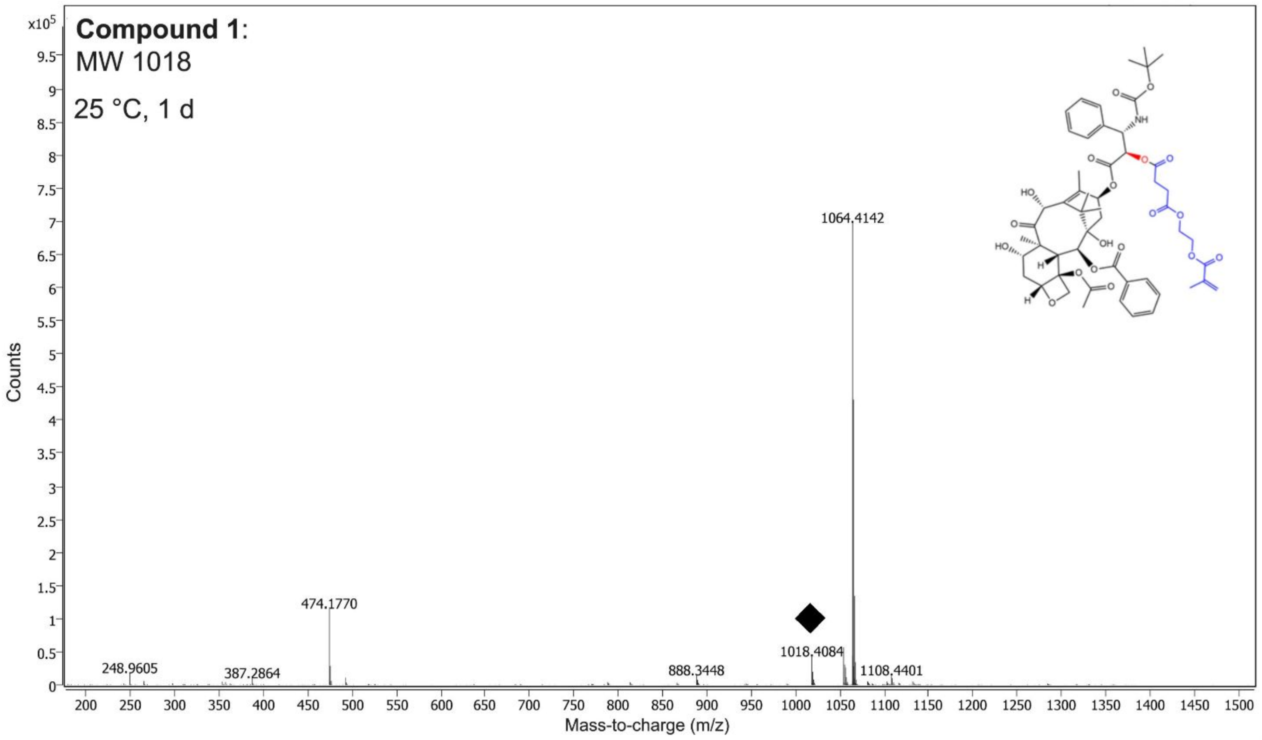

Figure S. 20: LC-ESI-MS spectrum and chemical structure of compound **1**; molecular ion theoretical mass for $[M_2 - H]^-$: 1018.4078. The molecular ion is indicated with black diamond. $M_2 = C_{53}H_{65}NO_{19}$.

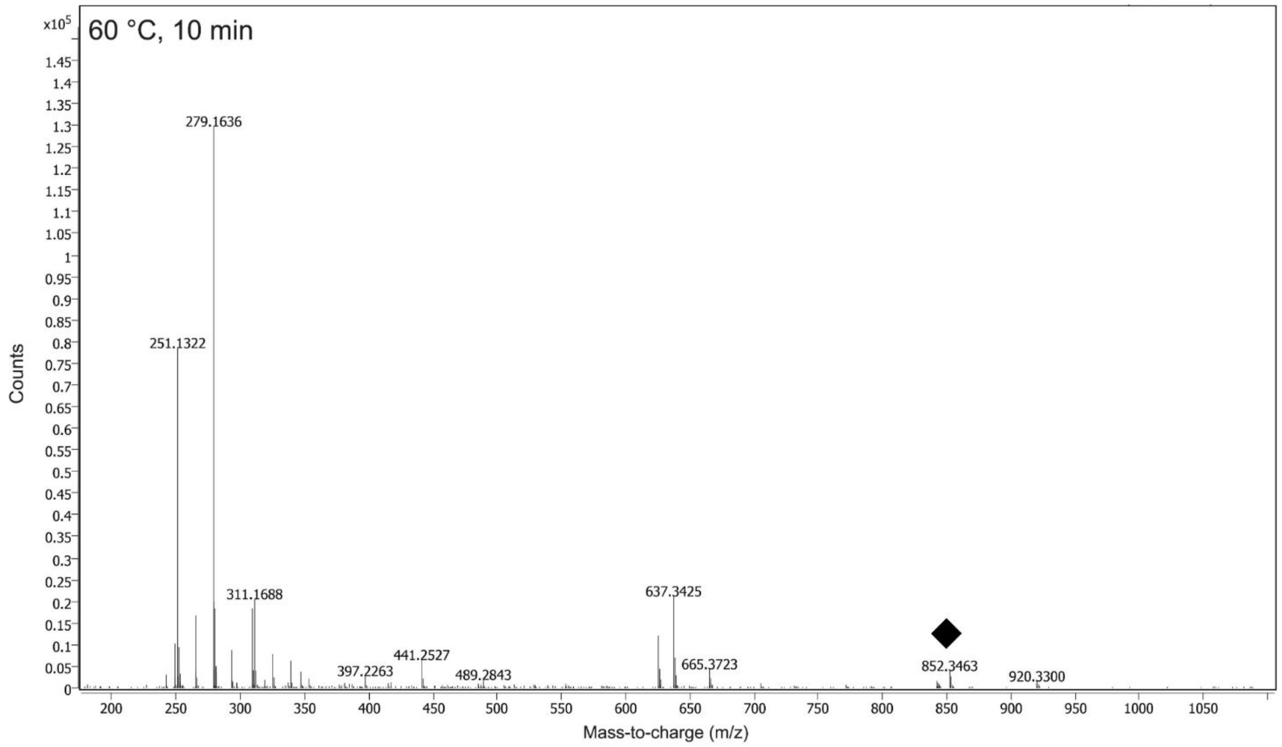

Figure S. 21: LC-ESI-MS spectrum of DTX released by NG@TEGMA_DTX_covalent at 60 °C after 10 min, molecular ion theoretical mass for [M + COOH]-: 852.3448. The molecular ion is indicated with black diamond. M = $C_{43}H_{53}NO_{14}$.

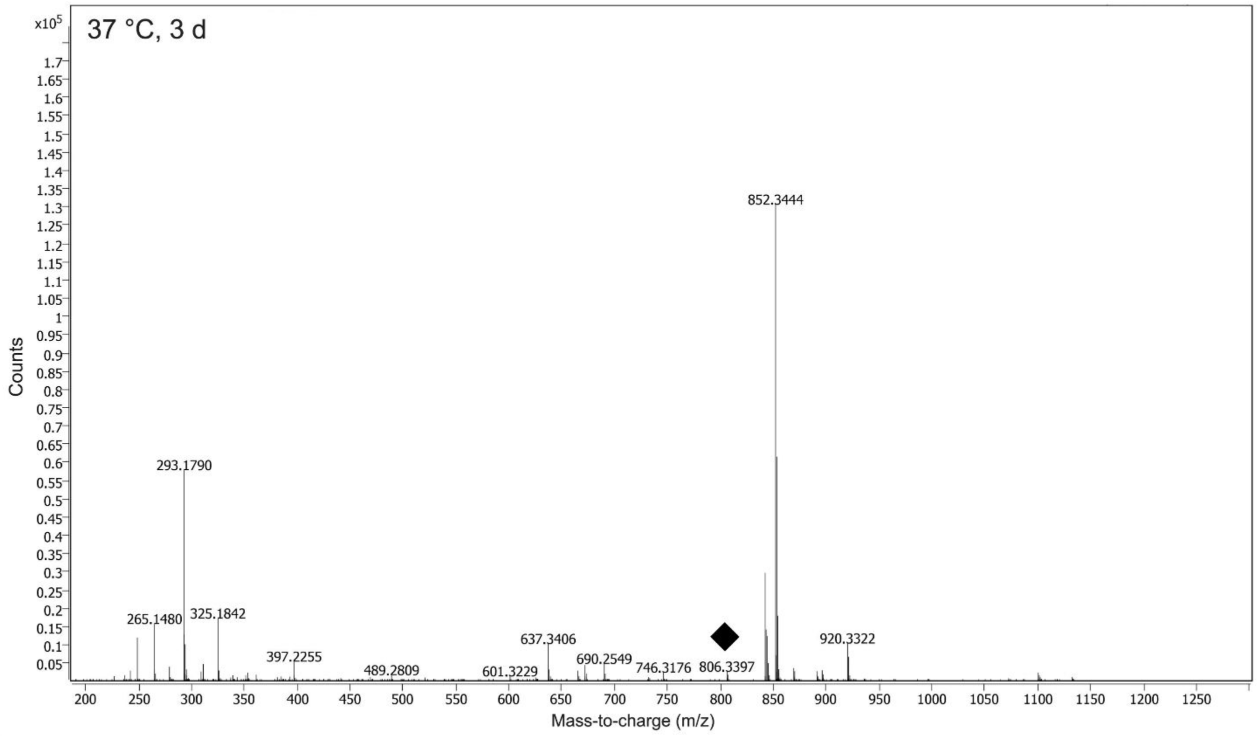

Figure S. 22: LC-ESI-MS spectrum of DTX released by NG@TEGMA_DTX_covalent at 37 °C at 3 d, molecular ion theoretical mass for [M - H]-: 806.3393. The molecular ion is indicated with black diamond. M = $C_{43}H_{53}NO_{14}$.

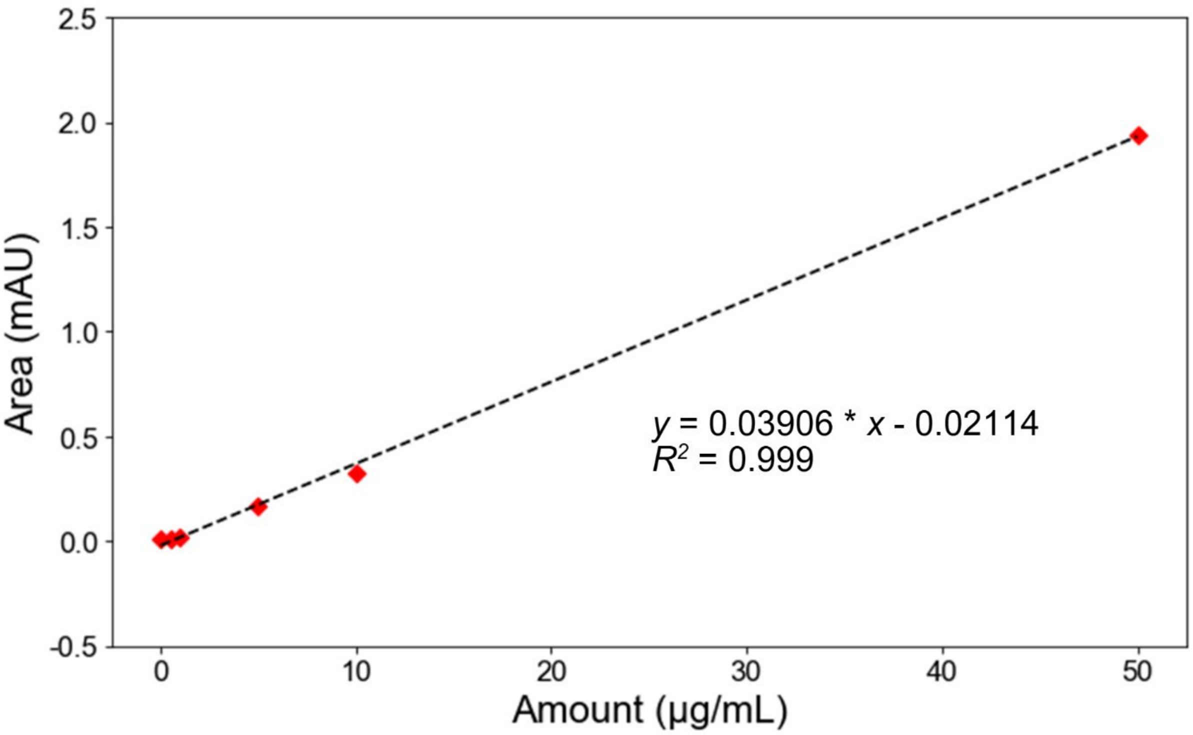

Figure S. 23: HPLC drug calibration curve for DTX according to the proposed method. Standards used (μg/mL): 0, 0.5, 1, 5, 10, 50.

References:

1. Hong, L.; Zhang, Z.; Zhang, Y.; Zhang, W. Synthesis and self-assembly of stimuli-responsive amphiphilic block copolymers based on polyhedral oligomeric silsesquioxane**.** *J. Polym. Sci. Part A: Polym. Chem*. **2014**, *52*, 2669–2683.

2. Van Tilburg, E. W.; Franssen, E. J. F.; van der Hoeven, J. J. M.; van der Meij, M.; Elshove, D.; Lammertsma, A. A.; Windhorst, A. D. Radiosynthesis of [^{11}C]docetaxel. *J. Label. Compd. Radiopharm*. **2004**, *47*, 763–777.

3. Borges, F. T. P.; Papavasiliou, G.; Teymour, F. Characterizing the Molecular Architecture of Hydrogels and Crosslinked Polymer Networks beyond Flory–Rehner—I. Theory. *Biomacromolecules* **2020**, *21* (12), 5104–5118.

4. Padmavathi, N.C.; Chatterji, P.R. Structural Characteristics and Swelling Behavior of Poly(ethylene glycol) Diacrylate Hydrogels. *Macromolecules* **1996**, *29* (6), 1976–1979.

5. Scotti, A.; Schulte, M.F.; Lopez, C.G.; Crassous, J.J.; Bochenek, S.; Richtering, W. How Softness Matters in Soft Nanogels and Nanogel Assemblies. *Chem. Rev.* **2022**, *122* (13), 11675–11700.
